# Novel Azo dyes containing a hydrazide-hydrazone moiety for dyeing polyester fabric

**DOI:** 10.1038/s41598-024-83565-3

**Published:** 2025-02-05

**Authors:** Ali A. Ali, Mostafa A. Ismail, Walid E. Elgammal, Amany Belal, Ahmed J. Obaidullah, Ali Kh. Khalil, Gameel A. M. Elhagali, Mohamed S. A. El-Gaby

**Affiliations:** 1 Department of Chemistry, Faculty of Science (Boys), Al-Azhar University, Nasr City, Cairo, 11884 Egypt; 2 Department of Chemistry, College of Science, King Khalid University, P. O. Box: 9004, 61413 Abha, Saudi Arabia; 3 Department of Pharmaceutical Chemistry, College of Pharmacy, Taif University, P.O. Box: 11099, 21944 Taif, Saudi Arabia; 4 Department of Pharmaceutical Chemistry, College of Pharmacy, King Saud University, P.O. Box 2457, 11451 Riyadh, Saudi Arabia; 5 Department of Chemistry, Faculty of Science, Ain Shams University, Cairo, 1156 Egypt

**Keywords:** Polyester, Synthesis, Dyes, Hydrazone, Benzophenone, Chemistry, Materials science

## Abstract

Novel azo dyes containing a hydrazide-hydrazone moiety linked to the benzothiazole nucleus are synthesized effectively in this study. The primary purpose of the study was to identify the best dyeing parameters, such as shade, temperature, pH, and time, in order to better understand the behaviour of dispersed dyes during polyester dyeing. To achieve excellent colour strength in value (K/S = 26), the ideal dyeing conditions for disperse dye **4** were 30 min, pH of 8, and 110 °C at shade 3%. While the ideal dyeing conditions for disperse dyes dye **10 **and dye **11 **were 30 min, pH of 2, and 130 °C at a shade of 3%. Depending on the coupler moieties, the coloured polyester samples ranged in hue from beige to dark brown. Furthermore, the impact of substituent’s was examined in relation to colour strength (K/S) measures and colourimetric coordinates (CIEL*a*b*) of dyed polyester fabrics. The synthesized dispersed dyes are good options for giving polyester textiles a variety of stable hues and very good colour strength as well as exceptional fastness to light, washing, and crocking.

## Introduction

Apart from the textile industry, polyester has extensive applications in diverse sectors such as packaging, automotive, construction, home products, and so forth. PET fabrics are a unique asset to the textile industry, providing outstanding chemical resistance, quick drying periods, dimensional stability, and high tensile strength at an affordable price^1^. Because of its hydrophobic qualities, polyester dyes so slowly when employing traditional dyeing techniques. Numerous characteristics of polyester, such as its robust structure, high degree of crystallinity, and lack of chemically active groups, are responsible for this^2^.

PET fibres can be dyed using disperse dyes, and the dyeing process is accomplished by dye molecules diffusing into the polymer. Nevertheless, the mobility of the polymer chain segments must produce sufficient volume or free space for the diffusion to occur before dispersed dye molecules may enter the polyester structure, which usually occurs at very high temperatures^3,4^. This is a result of polymer chains being able to move more freely in amorphous regions due to higher dyeing temperatures. Given that polyester dyeing is a physical process, the most important factors to consider when selecting a dyeing temperature are unquestionably the size and molecular weight of the dispersed colours^5^.

In recent years, experts have been focusing on the development of sustainable dyeing techniques, such as supercritical carbon dioxide, air dye, and plasma processing, in the textile sector^6^. Ultrasonic cavitation has been proven to improve both the dyeing rate constant and the dye absorbed by the material^7^. Recently, it has been possible to dye cotton fibre with an eco-friendly alternative hydrophobic organic chemical/water two-phase system that produces no effluent and no salt, achieving 71.43% dye reduction, above 98% dye uptake, and a 90% fixing rate^8^. The silicone oil emulsion dyeing system and silicone nonaqueous medium have recently shown success, indicating that this dyeing technology will transform the current traditional water dyeing process in terms of the significant problem of wastewater pollution and the enormous amount of water usage^9,10^. Using a nonaqueous medium with little water (NMLW) is a new reactive dyeing technique that was just created. Cotton textiles showed better colour depth than conventional water-based dyeing methods, as long as reactive dye fixing and dye uptake rates were higher^11^. Recently, it has been explained that cotton fibres can be reactively dyed without salt in a non-aqueous medium dyeing bath, significantly lowering the difficulty of wastewater treatment^12,13^.

One of the most important categories of organic colourants is azo-dyes and pigments, owing to their broad spectrum of applications in numerous sectors^14,15^. The nitrogen-nitrogen double bond that characterises azo dyes is an established characteristic that confers several benefits to the textile sector^16^. Conventionally, azo dyes are used to impart colour to a wide range of goods, such as paper, food, clothes, cosmetics, the pharmaceutical industry, and a lot more^17^. The hue of the dye is influenced by its wavelength absorption in the visible region, conjugation system, chromophore, and auxochrome group found within the dye molecule^18^. The title chemical is traditionally created from diazonium salt in combination with one or more electron-rich nucleophile segments^19,20^. The aromatic or heterocyclic amine initially transforms into a diazonium salt via the diazotization step^21^. The diazonium complex that results from the typical diazotization reaction interacts alongside various diazo-coupling nucleophile ingredients such as phenol, naphthol, or amine at low temperatures in the presence of salts and acids^22^.

Recently, other approaches to generating azo dyes involve reducing nitroaromatic derivatives in an alkaline medium, reducing nitroso compounds with AlLiH₄, oxidizing primary amines with lead tetraacetate or potassium permanganate, condensing hydrazines and quinones, as well as condensing primary amines with nitroso derivatives^16,17^.

An examination of the literature showed that the use of azo dye derivatives containing heterocycles is generally advantageous for the pharmaceutical industry and for the development of novel drugs^23^, and the dyeing industry^24–26^. The synthesis of heterocycle-containing azo dyes and their derivatives has attracted particular interest in recent years due to their potent biological activities as antimicrobial, antifungal, antiviral, anticonvulsant, antidiabetic, anti-inflammatory, antitubercular, anticancer DNA-binding, analgesic properties, and chemosensing tasks^27^. Based on an overview of the scientific literature, numerous azo dyes with heterocycles and their derivatives have been developed, and their potential applications in healthcare have also been disclosed^14^.

Benzophenone is an organic molecule that plays a crucial role as both a UV filter and a UV-ink photoinitiator^28,29^. It finds utility as a flavouring component, flavour enhancer, and perfume fixative in the perfumery business, along with its role as a reagent in organic chemistry^30^. It is also used as an ingredient in the production of adhesives, coatings, and plastics. Moreover, it is employed in the textile sector to shield materials against UV-induced deterioration^31^.

Peptide-protein connections have been widely recognised and mapped using benzophenones as photophysical probes^32^. It was discovered that a group of benzophenone materials were very effective antimalarial drugs against Plasmodium berghei in mice^33^. There is a vast array of biological functions associated with benzophenone derivatives, comprising antibacterial, antifungal, anti-carcinogenic, anti-HIV^34^, antioxidant^35^, anticancer^36^, anti-inflammatory^37^, and urease inhibitory^38,39^ properties39. Moreover, benzophenone hydrazone compounds have excellent insecticidal activity^40^.

Phenols are fundamental aromatic building blocks that are widely used in the synthesis of materials with beneficial properties, such as resins, natural products, active pharmaceutical ingredients, agrochemicals, dyes, tastes, and components that form fragrances^41,42^. In medicinal chemistry, the hydroxyl groups found within phenolic compounds have been assigned a prominent role owing to their biological behaviours comprising antioxidant, antiviral, antimicrobial, insecticidal, anti-sclerotic, anti-parasitic, and hypoglycemic properties^43,44^.

Resorcinol is an intriguing aromatic molecule that has been strategically incorporated into a variety of unique drugs to enhance their pharmacological profiles^45^. The aromatic ring structure within the resorcinol molecule has two hydroxyl groups that are positioned in relation to each other at their meta-positions. Resorcinol is a crucial ingredient in a variety of sectors, such as the tyre industry^46^, organic synthesis^47^, the field of dermatology^48^, wood bonding^49^, hair dye^50^, and drugs^51^. Additionally, resorcinol is present in flavonoids as the A-ring^52^ and in fermenting bacteria^53^. There are considerable toxicity risks associated with resorcinol use for both the environment and the health of humans. It is readily released into the environment and is highly soluble in water. When resorcinol is administered locally and taken orally, it might cause an aberration in the functioning of the thyroid gland, red blood cells, and central nervous system^54^. Pregnant women who consume resorcinol run the possibility of the foetus dying through respiratory failure^55^. Furthermore, resorcinol, which is made up of phenolic groups, might enhance the mechanical features of the film by giving polymers an opportunity to develop intermolecular hydrogen bond connections with each other^56^.

An organic molecule with hydrazone functionality linked to a carbonyl group is designated as a hydrazide-hydrazone^57^. In the design of novel drugs, this function constitutes a significant class of molecules. It is well-recognized that the active pharmacophoric group and the effect of hydrazide-hydrazones are linked. Derivatives of hydrazide-hydrazone have found widespread application in drug development, supramolecular assemblies, chemical synthesis, molecular switches, and molecular sensing^58–62^. The reality that nitrofurazone, furazolidone, and nitrofurantoin are all utilized as chemotherapy drugs that contain a hydrazide-hydrazone moiety ought to be emphasized^63^.

A novel category of azo dyes has been developed here by incorporating a hydrazide-hydrazone moiety linked to the benzothiazole nucleus, in keeping with our investigations on the insertion of the heterocyclic moiety into the azo dye scaffold. The molecular structures of newly generated azo dye molecules have been elucidated using spectroscopic techniques and elemental analysis. Furthermore, through the process of dyeing the prepared dyestuffs onto polyester fabrics, we will assess the colour intensity qualities, CLogP computation, spectral properties (light, crock, and washing), fastness, colour strength properties (K/S), auxochrome effect, and colour position in colourimetric coordinates (CIEL*a*b*).

## Results and discussions

### Organic synthesis

**Fig. 1:**
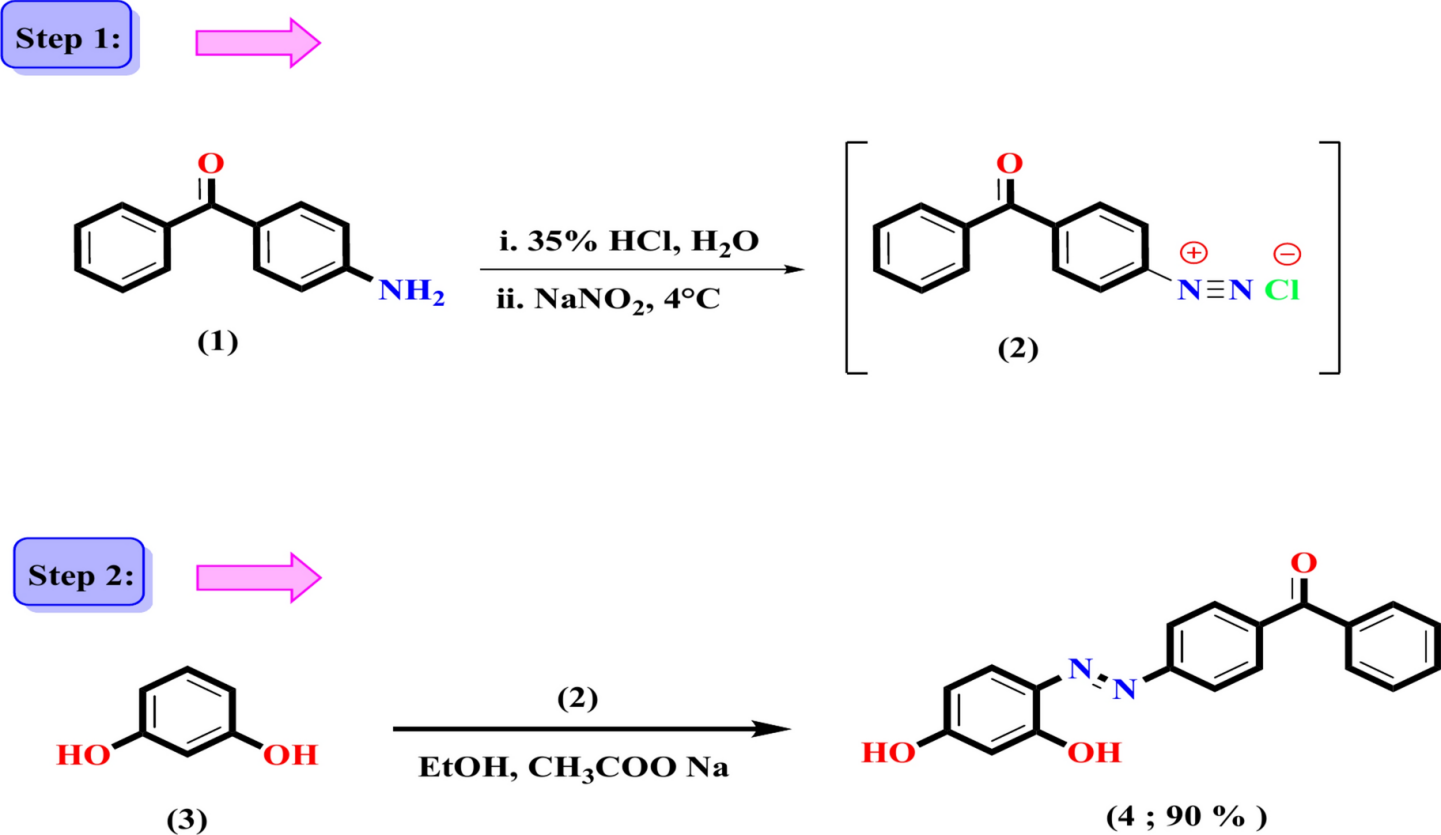
Generation of a novel azodye (4-(phenyl)diazenyl)(2,4-dihydroxyphenyl)methanone **4.**

**Fig. 2:**
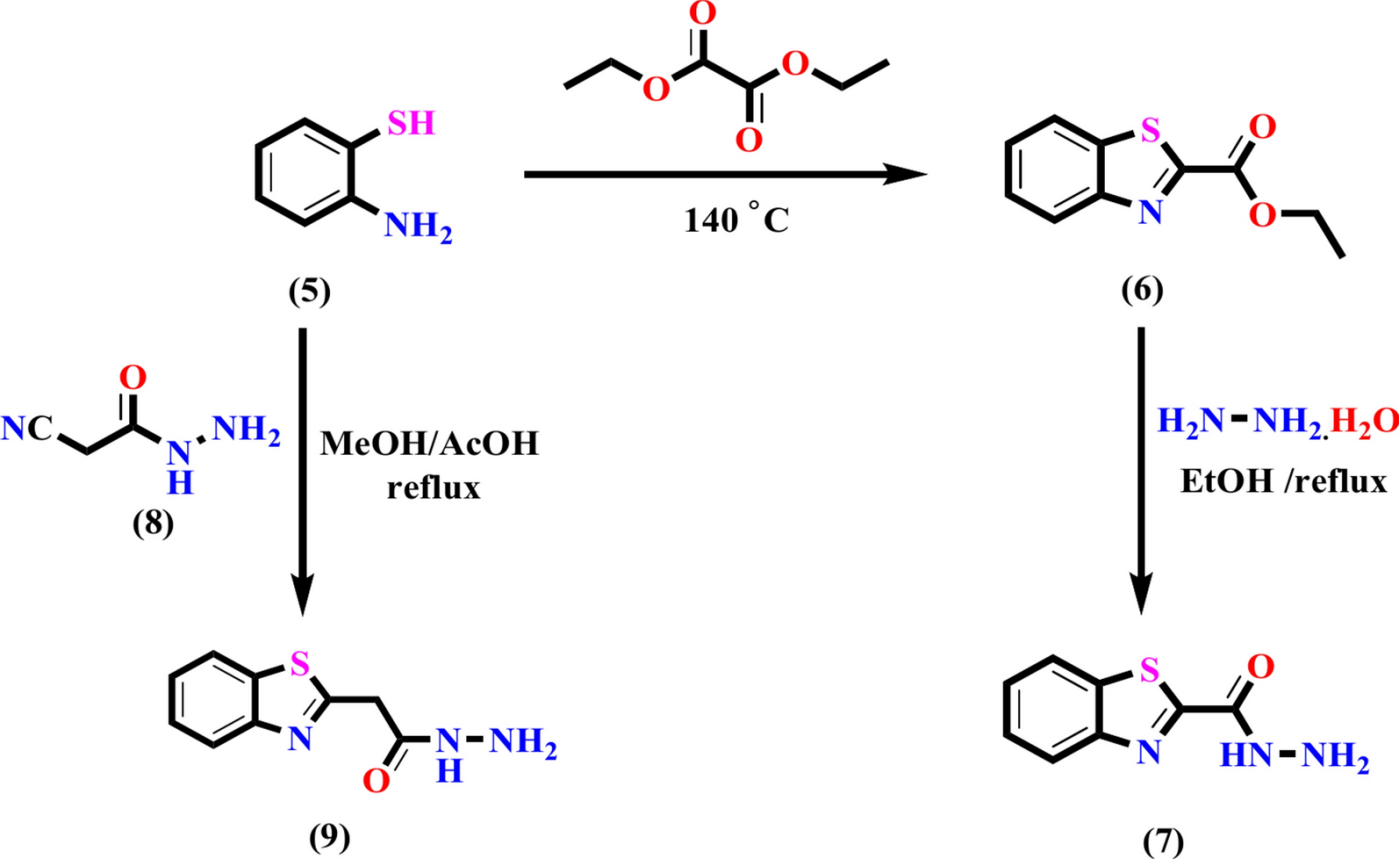
The synthesis of two crucial intermediates, benzothiazole-2-carbohydrazide **7** and benzothiazole-2-acetohydrazide **9.**

The synthetic approach utilized in this study for the target azo dyes (**4**, **6**, and** 8**) is illustrated in Figs. 1 and 2. The novel required ingredient, (*E*)-(4-((2,4-dihydroxyphenyl)diazenyl)phenyl)(phenyl)methanone **4**, has been generated via treating resorcinol **3** with diazotized 4-aminobenzophenone **1** in ethanol at a temperature between 0 and 5 °C. The product that was isolated achieved a yield of 90% and had been identified by physical attributes such as colour and melting point. The generated azo dye was refined by re-crystallisation, and TLC was applied to monitor the reaction and ensure its purity. The chemical structure of the isolated product was identified utilising elemental analysis and a number of spectral techniques, including Fourier transform infrared (FT-IR), ^1^H NMR, and ^13^C NMR. The infrared spectrum of compound **4** showed a band at 1623 cm⁻^1^, which is attributed to the carbonyl group stretching of the benzophenone moiety.The absorption bands observed at 3278 and 1567 cm^−1^ are due to phenolic and azo dye group stretching vibrations, respectively. The ^1^H-NMR spectrum of compound **4** (in DMSO-*d*_6_) displayed two doublets of a doubletat 6.51 δ (*J* = 8.9 and 2.5 Hz) and 7.57 δ (*J* = 8.2 and 6.9 Hz) along with a doublet at 6.37 δ (*J* = 2.5 Hz) that was triggered by the proton’s resorcinol fragment. A multiplet at δ_H_ = 7.65–8.00 ppm was attributed to the aromatic protons, whereas two downfield signals at δ_H_ = 10.74 and 12.36 ppm, which are exchangeable by D_2_O, were identified as belonging to two hydroxyl protons. The ^13^C NMR analysis additionally demonstrated the presence of a downfield signal at δ_C_ = 195.51 ppm for the carbonyl group situated between two phenyl moieties and the appearance of signals at δ_C_ = 103.49, 110.21, 122.04, 129.09, 129.88, 130.05, 131.51, 133.24, 133.39, 137.74, 153.48, 158.09, and 164.56. The mass spectrum presented further evidence for the coupling product that confirmed its molecular formula, C_19_H_14_N_2_O_3_ (M^+^;10.16%). The existence of an OH-phenolic group was demonstrated via a positive reaction to the FeCl_3_ test, which provided chemical evidence for the structure of **4**. The corresponding non-isolable reactive intermediate, diazonium chloride **2**, was generated via diazotizing the amino benzophenone **1** with hydrochloric acid and sodium nitrite solutions while it was being cooled with ice, as depicted in Fig. 1. The electrophilic diazonium salt **2** was coupled with the electron-rich component **3** at position 4 in the subsequent step. The two hydroxyl groups in the aromatic ring structure of the resorcinol molecule are situated in relation to each other at their meta positions. The high reactivity of resorcinol is mainly caused by the location of both of those hydroxyl groups within the benzene ring. Regarding the reactivity of resorcinol, the hydrogen atoms at the locations of carbon 2, 4, and 6, which are adjacent to the hydroxyl groups, are more reactive. Since the hydrogen atom at the resorcinol molecule 5-position is basically nonreactive, it is not involved in any chemical reactions under typical reaction situations. Electrophilic reactions with resorcinol are expected to occur readily at the 4- and 6-positions due to substitutions in phenols beginning at the more reactive para position rather than the ortho position^64^.

**Fig. 3:**
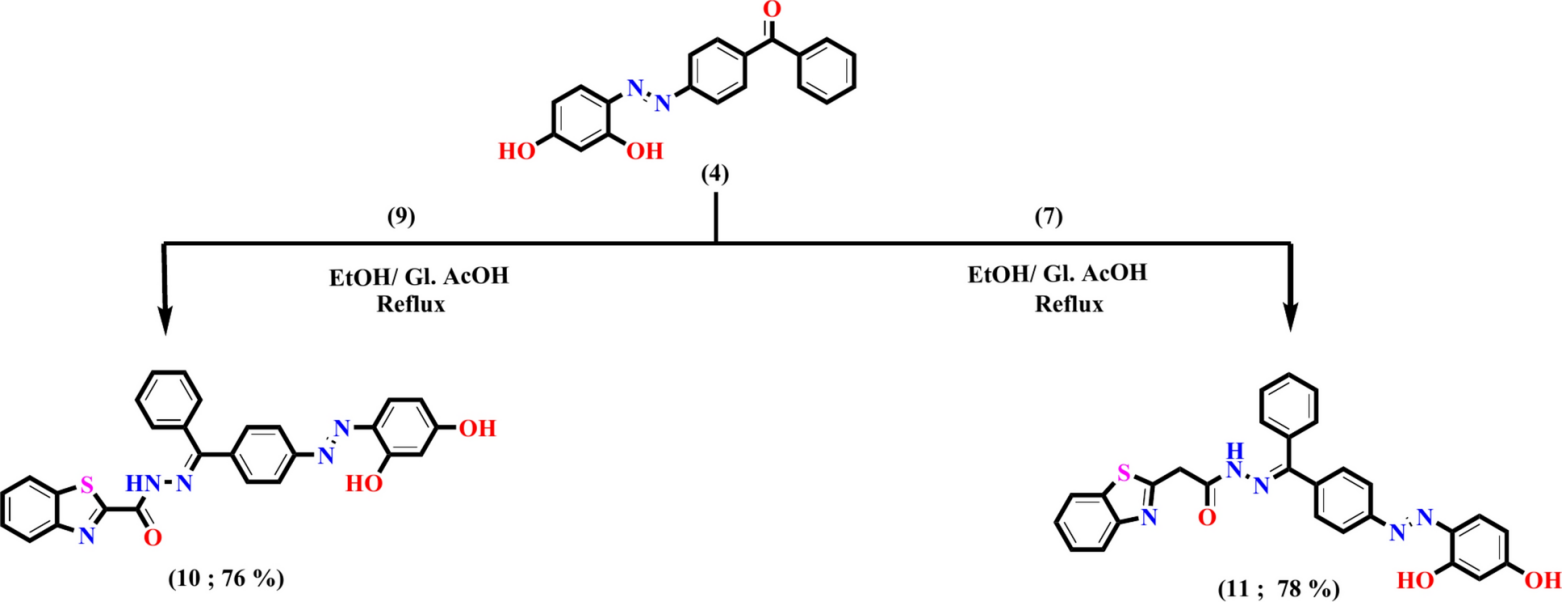
The synthesis of novel azo dyes **4**, **10** and **11** has a hydrazide-hydrazone moiety linked to the benzothiazole nucleus.

The bioactive features of the target derivatives have been enhanced by the insertion of the heterocyclic moiety into the azo dye scaffold. By incorporating heterocyclic moieties, drugs can easily be tailored for a variety of biological and pharmacological objectives. The effective approach designed for creating novel azo dyes **10** and **11**, which contain a hydrazide-hydrazone moiety linked to the benzothiazole nucleus, is illustrated in Fig. 3. The critical intermediates are benzothiazole-2-carbohydrazide **7** and benzothiazole-2-acetohydrazide **9** were initially created in accordance with the described procedure with the goal of synthesizing the suggested azo dyes. The cyclization of 2-aminothiophenol **5** with diethyl oxalate generated ethyl benzothiazole-2-carboxylate **6**, which subsequently reacted with hydrazine to yield the target hydrazide **7**, as depicted in Fig. 2^65^. The synthesis of benzothiazol-2-ylacetohydrazide **9** in one step involves the cyclocondensation of 2-aminothiophenol **5** with cyanoacetohydrazide**8** by refluxing in methanol and acetic acid as the reaction media used. The synthesis of the novel Azo Dye**10**, which contains a hydrazide-hydrazone moiety, involved condensation of carbohydrazide 7 with benzophenone derivative **4** by refluxing in ethanol and a catalytic quantity of glacial acetic acid with a reaction yield of 76%. The presence of imine and carbonyl groups was confirmed through absorption bands in the infrared spectrum at 1649 and 1632 cm^−1^, respectively. In addition, two absorption bands at 3381 and 1551 cm^−1^ were observed in the infrared spectra, which indicated the existence of OH and N=N groups, respectively. Two downfield signals at δ_H_ = 10.41 and 12.20 ppm, which are exchangeable by D_2_O and represent NH and two hydroxyl protons, respectively, have been observed in the ^1^H NMR spectrum of compound **10**. Also, the ^1^HNMR spectrum of compound **10** revealed two doublets of a doublet at δ_H_ = 6.51 (*J* = 8.9 and 2.5 Hz) and 8.18 ppm (*J* = 8.2 and 6.9 Hz), as well as a doublet at δ_H_ = 6.37 (*J* = 2.5 Hz) that was caused by the protons in the resorcinol moiety. On the other hand, the remaining aromatic protons are found in the range of 7.50–8.08 ppm. Two distinct signals were identified at δ_C_ = 164.04 and 159.29 ppm, respectively, in the ^13^C NMR spectrum of compound **10**, which corresponded to carbonyl and imine carbons. Additionally, the ^13^C NMR spectrum demonstrated signals at δ_C_ = 103.50, 110.28, 122.00, 123.32, 124.35, 127.16, 127.50, 129.09, 130.04, 130.63, 131.51, 132.70, 133.25, 133.37, 136.11, 137.42, 150.32, 152.15, 153.24, 153.43, 158.09, and 158.60 ppm, which correspond to various types of carbon atoms present in the compound. A molecular ion peak was observed in the mass spectrum of compound **10** at m/z = 493 (10.23%), which matches the molecular formula of C_27_H_19_N_5_O_3_S. The presence of an OH-phenolic group was proven through a positive FeCl_3_ test outcome, which offered chemical support for the structure of **10**. It has been suggested that compound **10** forms as a result of the nucleophilic attack of acid hydrazide **7** on the carbonyl group of compound **4** and the subsequent removal of water molecules. The formation of compound **11** was obtained through the same procedure, switching carbohydrazide **7** with acetohydrazide** 9**. The infrared spectrum of this compound revealed absorption bands at 3188, 1647, 1618, and 1551 cm^−1^, which were recognized as hydrazone N–H, C=O, C=N, and N=N groups, respectively. The ^1^HNMR spectrum of compound **11** displayed a distinctive signal for methylene protons at 4.13 ppm, as well as three downfield signals observed at δ_H_ = 10.55, 10.73, and 12.37 ppm, which are exchangeable by D_2_O and are associated with NH and two hydroxyl protons, respectively. Furthermore, two doublets of a doublet at δ_H_ = 6.51ppm (*J* = 8.8 and 2.5 Hz) and 7.87ppm (*J* = 8.3 and 6.9 Hz) were observed in the ^1^HNMR spectrum of compound **10**, in addition to a doublet at δ_H_ = 6.37 (*J* = 2.4 Hz) that was brought on by the protons in the resorcinol moiety. In the range of δ_H_ = 7.40 and 7.75 ppm, the remaining thirteen aromatic protons of the phenyl rings and benzothiazole moiety were evident. The ^13^C NMR spectrum of compound **11** revealed that the signal at δ_C_ = 38.39 ppm belongs to the methylene group distinct carbon and two downfield signals at δ_C_ = 168.70 and 175.49 ppm, which are connected to carbons of C=N and C=O, respectively. The mass spectrum verified compound **11**, which possessed the molecular ion peak at m/z = 507 (6.72%), matching its molecular formula C_28_H_21_N_5_O_3_S.

### Characterization of the synthesized dyes by UV–Vis

**Fig. 4:**
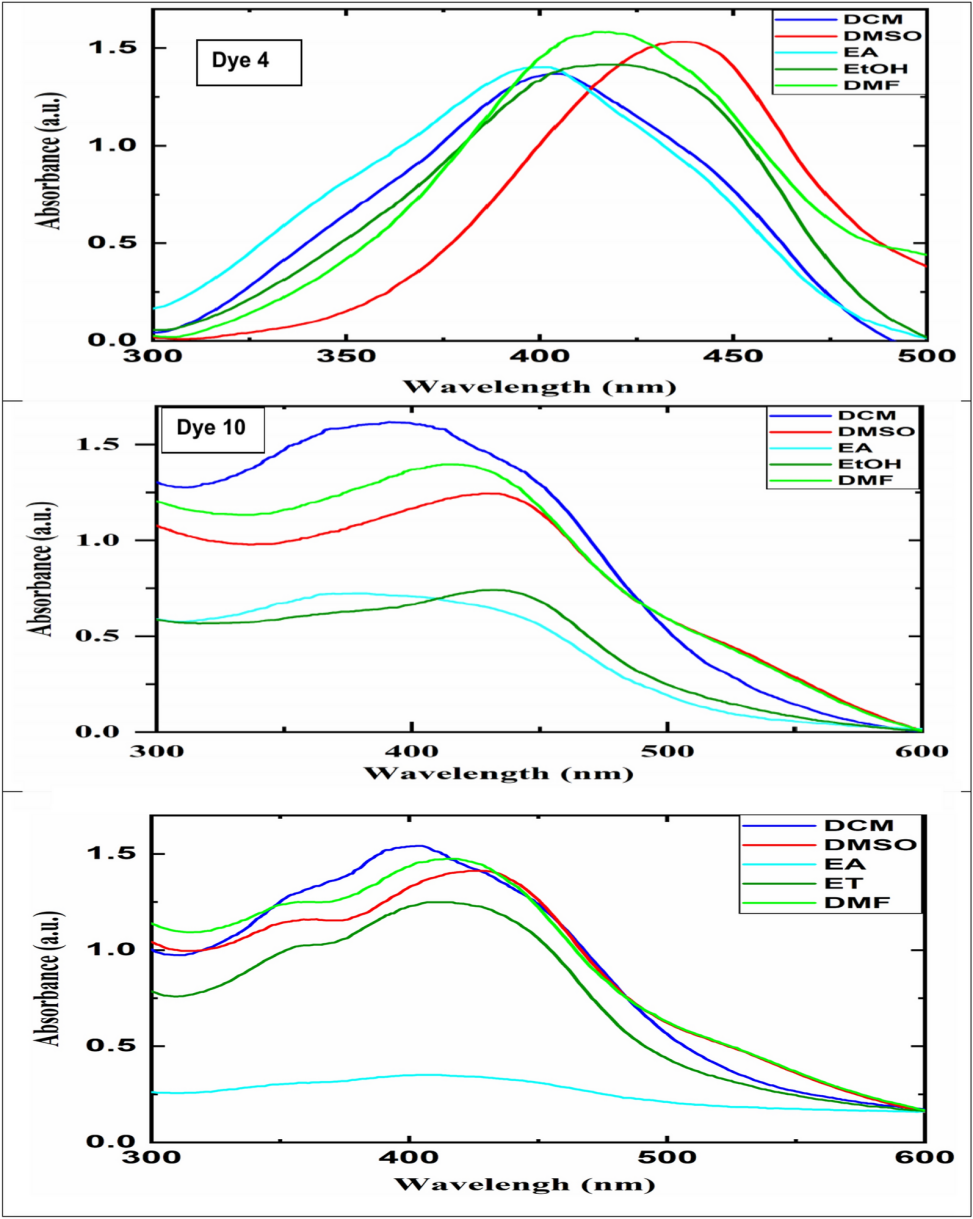
Dyes (**4**, **10**, and **11**) and their absorption spectra curves in various solvents (ethyl acetate (EA), Dimethyl sulfoxide (DMSO), dichloromethane (DCM), ethanol (ET), and Dimethylformamide (DMF).

Figure 4 depicts the visible absorption spectra of Dyes **4**, **10**, and **11** in five solvents (ethyl acetate, DMSO, dichloromethane, ethanol, and DMF) to investigate the effect of solvent polarity on the spectral characteristics at 4 × 10^–3^ mg/L. Table 1 lists the spectrum properties, including full widths at half maximum FWHM, λmax, and ε (molar absorption coefficients).The plots of absorbance (A) against concentration (C) gave the extinction coefficients (ε) equal to 96,443, 82,143, and 91,836 L mol^−1^ cm^−1^ for dyes **4**, **10**, and **11**, respectively, showing these dyes are appropriate for colouring polyester fibres (Beer-Lambert law, A = ε*C)^66^.

**Table 1:** The dyes’ (**4**, **10**, and **11**) photophysical characteristics in various solvents.

Solvent	FWHM			*λ*_*max*_ (nm)			Ɛ (L⋅mol^−1^ cm^−1^)		
	Dye **4**	Dye **10**	Dye **11**	Dye **4**	Dye **10**	Dye **11**	Dye **4**	Dye **10**	Dye **11**
DMF	139.63	259.31	247.30	415	416	416	80,612	70,918	80,612
DMSO	99.21	280.44	268.42	437	435	428	78,061	63,265	79,081
Ethyl acetate	114.55	264.09	255.36	401	369	410	74,489	36,072	12,755
Ethanol	106.13	269.58	266.53	417	433	412	71,938	37,755	71,938
DCM	110.80	264.61	230.45	404	390	404	70,408	82,143	91,836

As shown in Table 1, the polarity of the solvents (DMSO is more polar than other solvents) had a substantial effect on the maximum absorption wavelength and FWHMs of the new dyes. As a result, it is preferable to add a fraction of DMSO to the dye bath since it produces a bathochromic-shift effect for all dyes. This is because the polar solvent can stabilize the charge separation form in the excited state of dye molecules while also decreasing the energy of the excited state, resulting in a bathochromic-shift effect^67^. For the same reason, a more broad-absorbing peak may form as a result of the increase in solvent polarity, which may also enhance the change in the distance between the dye molecules’ nuclei before and after excitation. The FWHMs for the DMF and DMSO solvents were larger than those for the other three solvents^67^. Determining azo-hydrazide-hydrazone tautomerism is a crucial area of study, particularly for dye applications in dyeing industry and technology, as the two tautomeric structures have distinct technical, biological, and photophysical characteristics. Potential azo-hydrazone tautomers are present in the synthetic colours used in our investigation^68^.

### CLogP computation

The ChemDraw software’s CLogP server computations were used to determine the dyes’ ClogP (N-octanol/water partition coefficient) values. Disperse dyeing of polyester simply involves distributing disperse dyes between two immiscible solvents (water and polyester fibers). Consequently, the dye will migrate more to the hydrophobic polyester fibres if it is less soluble in the dyebath (i.e., more hydrophobic). Consequently, the ClogP values are discussed below. Lower ClogP generally indicates that the disperse dye is more soluble in the aqueous dyebath. The final K/S values are negatively correlated with the disperse dye’s solubility^69^. As a result, dyes **10** and **11** (which have the lowest solubility in an aqueous dye bath because of their higher ClogP values of 8.4 and 7.15, respectively) ought to exhibit greater K/S values than dye **4** (which has the highest solubility because of its lower ClogP value of 4.73).On the contrary, dyes **4**, **10**, and **11** exhibit the reverse trend; that is, dyes **10** and **11** with the highest ClogP values also have the lowest K/S values. In this instance, size may play a significant role, which could be explained by the dye molecules’ effective intrafibre diffusion within the fabrics^5,25^. As a result, even though dyes **10** and **11** exhibited greater ClogP values than dye **4**, it’s possible that dye** 4**, which is the smallest, diffused more in the polyester polymer matrix and so displayed higher K/S values.

### Characteristics of dye application on polyester fabrics

#### Depth of shade

Polyester fabrics were dyed using dispersing agents at temperatures between 100 and 130 °C for 10–60 min at different shading levels of 0.5, 1, 2, 3, 4, 5, and 6%. The polyester fabrics were dyed to create colour tones such as dark brown, beige, and brownish red.

#### Color strength measurements (K/S) and analyses

K/S values at the wavelengths of greatest absorbance at (410, 440, and 410) for dyes **4**, **10**, and **11**, respectively, or minimum reflectance, typically the behaviour of dye deposition on textile surfaces. It was discovered that all dyed fabrics **4**,** 10**, and **11** at various parameters had positive values for a* and b*, which denote a shift in the dye’s colour hues to a yellowish direction on the yellow-blue axis and a reddish direction on the red-green axis, respectively. All the synthetic dyes **4**, **10**, and **11** show that positive hue angle (H^o^) values indicate a shift towards a yellowish hue, while negative hue angle (H^o^) values show a shift towards a reddish hue. The amount of visible light that a dyed fabric reflects is indicated by its reflectance value. The reflectance value of white fabric samples is 100% because they reflect all light wavelengths, whereas the reflectance value of black fabric samples is 0% because they absorb all light wavelengths. As a result, all other colours’ reflectance levels lie somewhere between these two extremes. The chemical structure and substituent of the aromatic moiety of the dyes generated had an impact on the K/S values, leading to a range of dyeing values. In comparison to dyes **10** and **11,** dye **4** had a stronger hue, which produced superior results. This could be the effect of dyes **10** and **11**'s increased molecular weight due to their higher coupling of the azo benzophenone derivatives with the benzothiazole, which in turn causes more dye to disperse in the dyeing water due to their lower adsorption^70^.

#### Dyeing pH effect on reflectance (R%) and color strength (K/S)

**Fig. 5:**
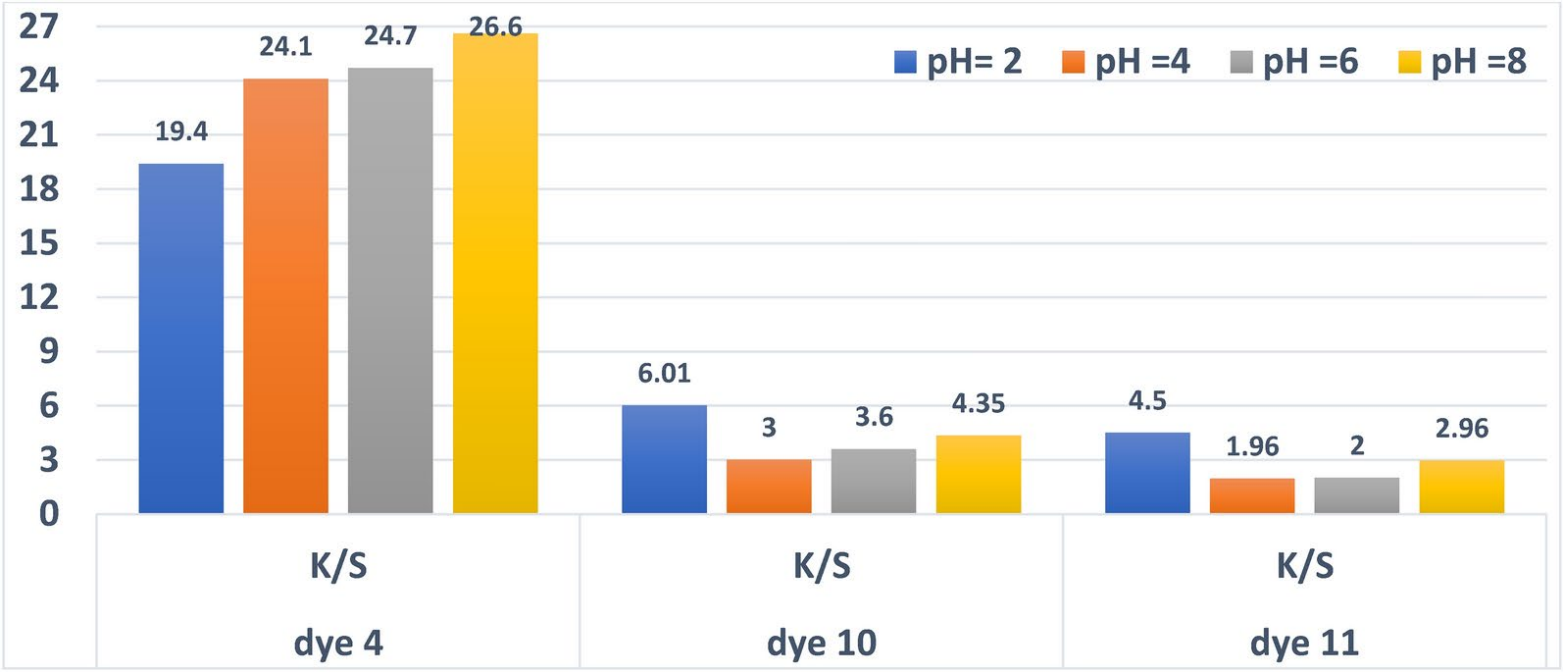
Relationship between pH and color strength for dyes **4**, **10** and **11** (time = 30 min, Temp. = 130 °C, shade = 3%).

As can be seen in (Fig. 5) for dyes **4**, **10** and **11**, different K/S were obtained across the acidic pH range (2, 4, 6, and 8). Additionally, it implies that the dyes under study dye in a way that is significantly influenced by variations in the dyeing pH. Increased dyeing pH above 2 resulted in lower K/S values and higher reflectance values for dyes **10** and **11**, as can be observed in (Fig. 5). It was found that dyes **10** and **11** had their strongest colour strength at pH 2, which was followed by pH 8, pH 6, and pH 4, in that order. Contrary to common knowledge, a pH of **4** is preferable to a pH of 2. Due to its small molecular weight, dye **4** had a far higher colour strength value (26.6) than the other two dyes, even though it had the best pH of 8, but often, polyester dyeing is done at an acidic pH, this might be because dye No. **4** becomes more soluble due to the reaction of two hydroxyl groups in an alkaline media, intensifying the colour intensity.

Azo dyes having OH functional groups in para positions relative to the –N=N– moiety undergo a tautomeric transition from enol/azo to keto/hydrazone when the solution’s pH shifts from acidic to basic. The tautomerism that occurs with azo compounds has significant effects on their hue, K/S, and physical features. Therefore, dyes No. **10** and **11** display two types of tautomerism, both the azo-hydrazone and keto-enol tautomerism phenomena, throughout all of their chemical configurations^71–73^. The results of this investigation indicate that the ideal pH for dyeing polyester with dye numbers (**10** and **11**) is 2, and the ideal pH for dyeing polyester with dye number (**4**) is 8 as can be observed in Fig. 5 and Table 2.

**Table 2:** a*, b*, L*, C*, E, H^o^ measurements for dyes **4,10** and **11** (time = 30 min, temperature = 130 °C, and 3% shade) are affected by dyeing pH.

Dye	pH	L*	a*	b*	C*	H^o^	E	Original pictures of the dyed fabrics
**4**	pH 2	64.4	23.8	71.5	75.4	71.6	99.1	
	pH 4	56.5	20.5	62.7	66	71.9	86.9	
	pH 6	53.4	20.8	58.8	52.4	70.6	82.1	
	pH 8	50.6	20.8	56	59.7	69.7	78.3	
**10**	pH 2	57.6	12	36.9	38.7	72	69.5	
	pH 4	64.2	7	33.9	34.6	78.4	72.9	
	pH 6	60.6	7.8	32.9	33.8	76.7	69.4	
	pH 8	59.3	9.5	35.5	36.7	75	69.8	
**11**	pH 2	58.5	9.3	31.6	33.0	73.6	67.1	
	pH 4	68.1	7.7	30.7	31.6	76	75.1	
	pH 6	67	8.1	29.4	30.5	74.6	73.6	
	pH 8	61.6	9.1	29.4	30.8	72.9	68.9	

#### Dyeing temperature’s effect on reflectance (R%) and color strength (K/S)

**Fig. 6:**
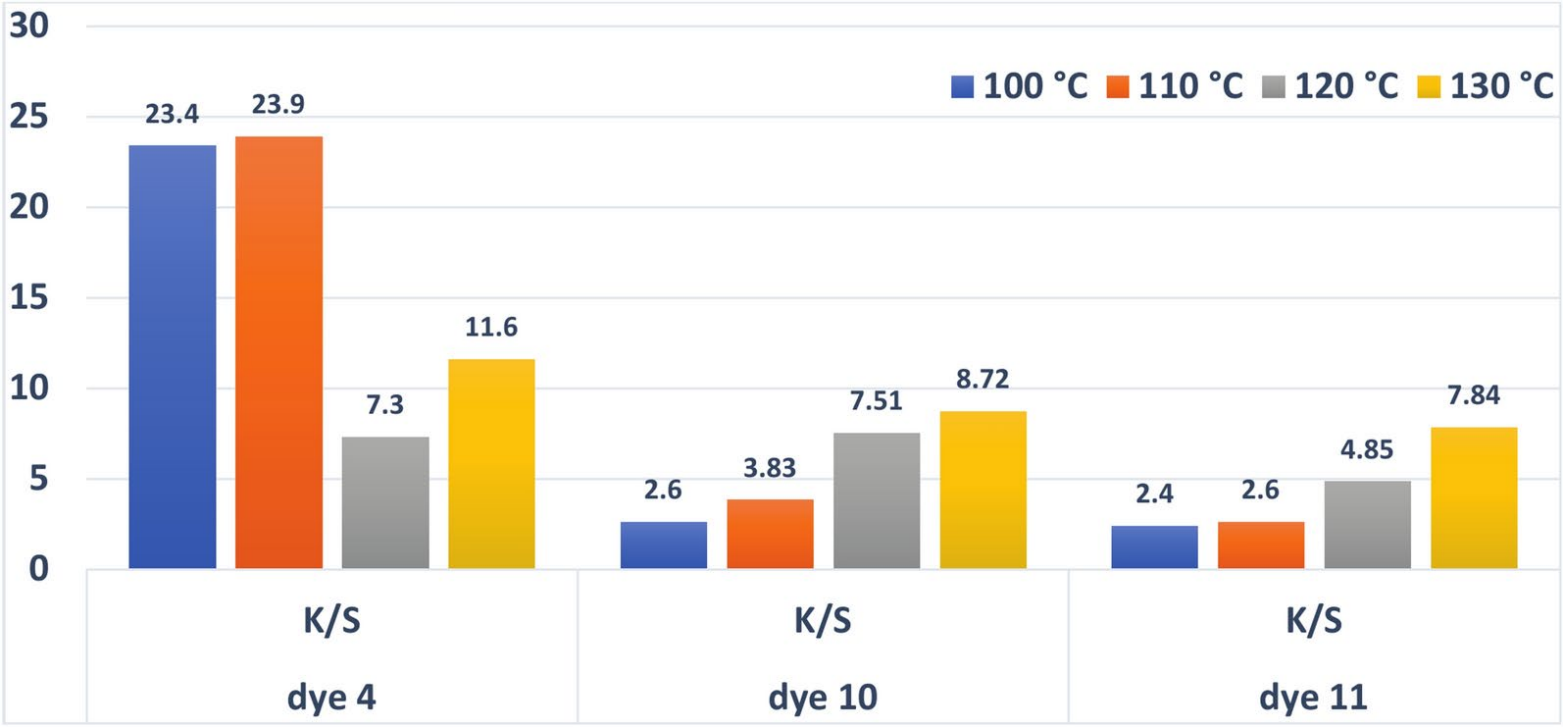
Relationship between color strength (K/S) and temperature for dyes **4**, **10** and **11** (time = 30 min, shade = 3 (pH = 2 for dyes **10** and **11** and pH = 8 for dye **4**)).

The temperature range for this procedure was 100–130 °C, and polyester samples were coloured using dispersed dyes. Figure 6 illustrates how temperature changes affect the colour strength of polyester dyed in comparison to traditional aqueous dyeing at 100 °C. Figure 6 shows that the colour strength of dyed polyester fabric increases with dye bath temperature up to equilibrium, after which it declines with increasing dyeing temperature. This change could be explained by the dye’s shifting balance from the fabric to the dye bath^6^. According to Fig. 6, the temperature increased from 100 to 130 °C, increasing the K/S for three dyes. This may be explained by the dye molecules’ high kinetic energy and high dispersion rate. As a result, 130 °C was determined to be the ideal temperature for the application. The dye may not transform into clothes above the optimal temperature, reversing the dyeing process and lowering the K/S and fastness qualities^74^.

Figure 6 shows that the optimal temperature for dyes **10** and **11** was 130 °C, with values of 8.72 and 7.84, respectively. This indicates that the ideal temperature for dispersing dyes 10 and 11 in water with a dispersing agent is the limitation temperature of 130 °C, at which equilibrium occurs. While the best temperature for dye 4 was found to be 110 °C, with a value of 23.9. This is because dye **4** is smaller than the other two dyes, **10** and **11**, in molecular weight. So, the temperature of dye No. **4** rises above 100 °C; it increases kinetic energy, which increases randomness and so reduces the colour’s strength. This implies that temperatures above 110 °C weakened the dye, which in turn weakened the colour power of case dye **4**. This suggests that 110 °C was the ideal temperature for dye **4** to function at. Additional details and observations regarding this variation may be found in Fig. 6 and Table 3.

**Table 3:** The dyeing temperatures of dyes **4**, **10** and **11** ((pH = 2 for dyes **10 **and **11** and pH = 8 for dye** 4**), time = 30 min, and 3% shade) have an impact on the measurements of L*, a*, b* E, H^o^, C*, R%, and color strength.

Dye	Temp	L*	a*	b*	C*	H^o^	E	Original pictures of the dyed fabrics
**4**	100°C	63.8	15	74	78.1	71.4	100.9	
	110°C	50.6	20.8	56	59.7	69.7	78.3	
	120°C	66.4	15	53.9	55.9	74.5	86.8	
	130°C	62.1	24.1	71.9	75.8	71.5	98.01	
**10**	100°C	69.3	8.1	38.1	39	78	79.49	
	110°C	62.5	9	36.8	37.9	76.5	73.1	
	120°C	57.6	18	46.9	50.3	72	76.5	
	130°C	53.9	12.8	38.1	40.2	71.5	67.23	
**11**	100°C	68.2	6.5	34	32.7	79.5	76.61	
	110°C	67.7	6.51	32.8	33.4	78.8	75.5	
	120°C	58.3	8.8	33.5	34.7	75.3	67.81	
	130°C	51.1	10	33	34.5	73.1	61.64	

#### Dyeing time’s effect on reflectance (R%) and color strength (K/S)

**Fig. 7:**
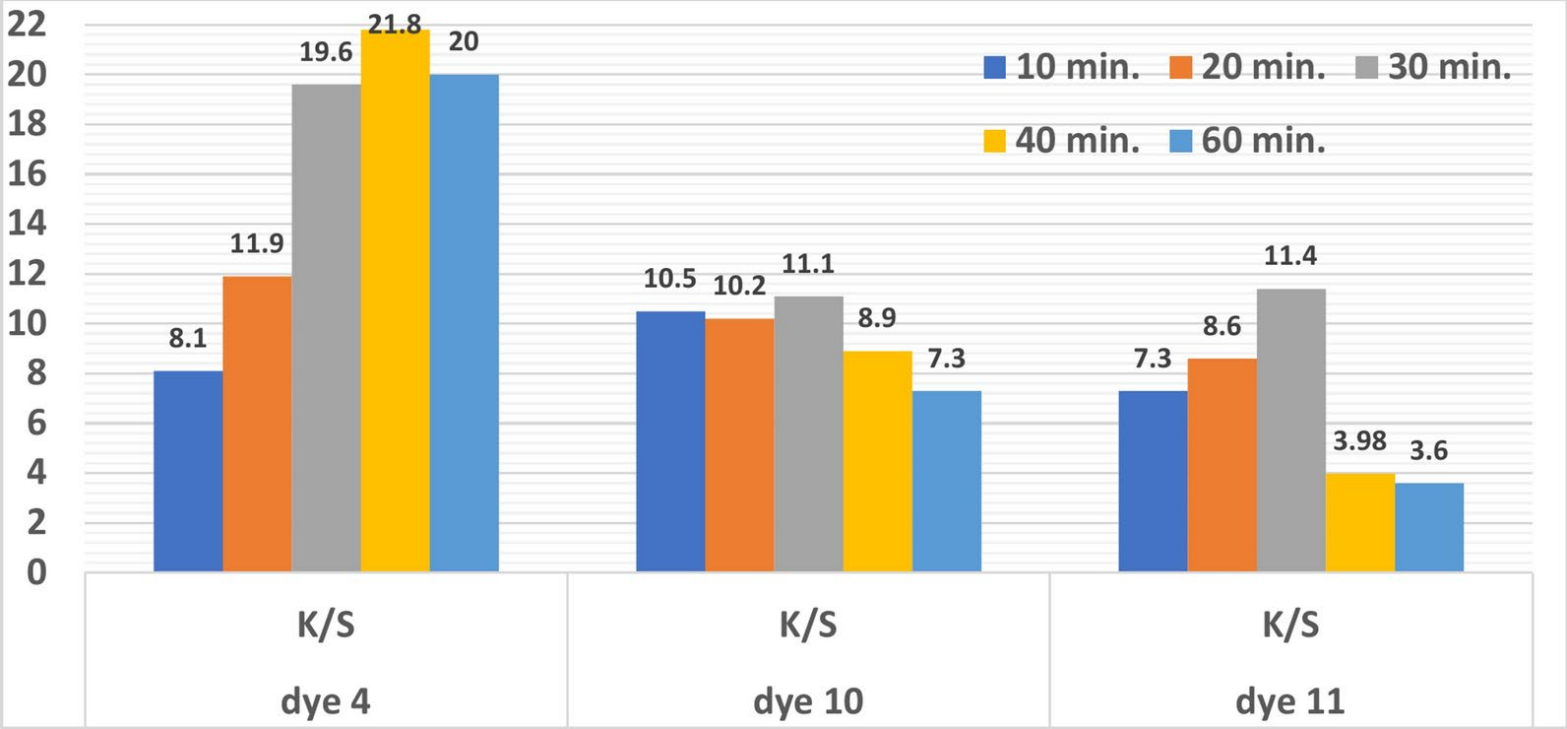
Relationship between color strength and time for dyes **4, 10** and **11** (Temp. = 130 °C for dyes **10** and **11** and Temp. = 110 °C for dye **4**), (pH = 2 for dyes **10 **and **11** and pH = 8 for dye **4**), shade = 3%).

The colour strength of dyed polyester for various dyeing time intervals ranging from 10 to 60 min is displayed in Fig. 7. The dyeing was done in an acidic pH of 2 for dyes **10** and **11** and a basic pH of 8 for dye **4** at 130 °C for varying times. A similar pattern to the influence of temperature was noted: until dyeing equilibrium is reached, colour strength increases as dyeing time increases. In the case of dyes **10** and **11**, further colour strength declines as dyeing duration increases from 30 to 40 and 60 min, because during this time the dye molecules inside the fibre and in the dye bath are randomly distributed^6^.

Figure 7 illustrates that dyes **10** and **11** had the best colour strength values (11.4 and 11.1) when dyed for 30 min, while the best colour strength value (21.8) for dye **4** was when dyed for 40 min. The amount of dye molecules in the dyeing solution and those within the fibre reached equilibrium after thirty minutes. When the application period was extended beyond thirty minutes, the K/S values decreased. This could be explained by the fact that equilibrium is favoured in the treatment bath, and prolonged heating could result in stripping^74^. This study’s findings indicate that dyeing polyester with two dyes (**10** and **11**) for 30 min is the ideal amount of time.

#### Dyeing shade’s effect on reflectance and color strength

**Fig. 8:**
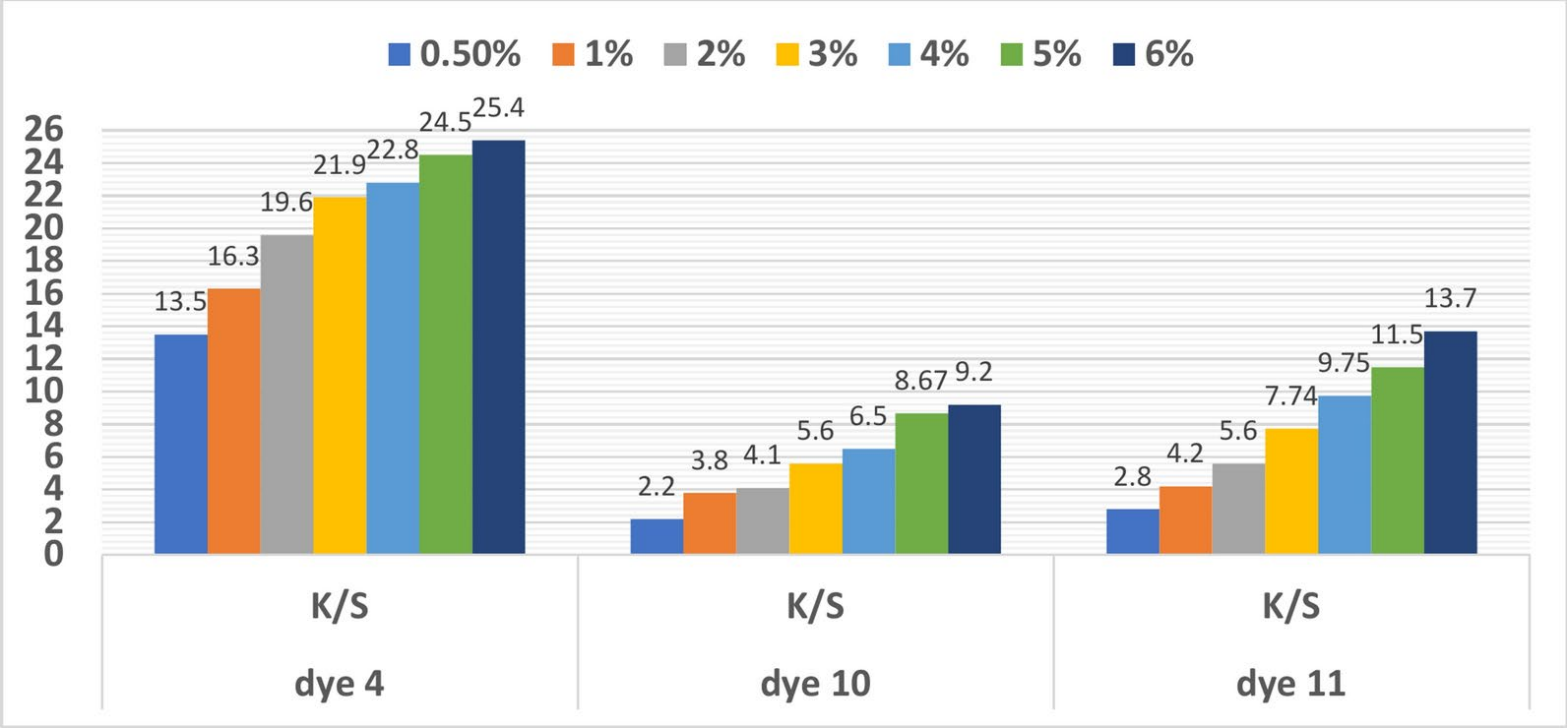
Relationship between color strength and shade for dyes **4**, **10** and **11** (Temp. = 130 °C for dyes **10** and and Temp. = 110 °C for dye ), (pH = 2 for dyes **10** and **11** and pH = 8 for dye **4**), (time = 30 min for dyes** 10** and 11 and time = 40 min for dye **4**).

Figure 8 presents the colour strength (K/S values) of polyester fabrics dyed in a variety of disperse dye hues: **4**, **10**, and **11**. Increasing the shade (0.5, 1, 2, 3, 4, 5, and 6% o.w.f.) while dyeing polyester resulted in better K/S values, indicating higher colour strength. We find that, in the instance of dye **4**, the increase in K/S value (3.5, 3.6, and 5.96 in value) is small from shade 3 to 6, as shown in Fig. 8. Shade 3 was therefore selected to be used in the earlier effects. This may be because the dye particles became saturated in the pores of the polyester materials, preventing further absorption.

For shade, 6% o.w.f., the greatest colour strength values achieved for disperse dyes **4, 10**, and **11** were 25.4, 9.2, and 13.7, respectively, as shown in (Fig. 8). At pH = 2 for dyes **10** and **11** and pH = 8 for dye **4**, 130 °C, and 30 min of dyeing time, these results were obtained. All three dyes’ reflectance ratings decreased as the shadow increased. As can be seen in Fig. 8, 6% was the perfect shade for all dyes, due to the high K/S ratio obtained. This implies that the increasing concentration of dye particles in the polyester led to increased absorption and decreased reflectance.

Based on Tables 2, 3, 4 and 5, it shows that dye No. **4** has greater (a*, b*, and C*) values than dye No. **10**, and dye No. **10** has higher values than dye No. **11**, which contradicts the values L* in all parameters (pH, temperature, time, and shade). This also shows that dye No. **4** has a higher K/S than dye No. **10**, followed by dye No. **11**.

**Table 4:** Dyeing periods have an impact on the measurements of L*, a*, C*, b*, E, H^o^, R%, and color strength for dyes **4, 10** and** 11** (Temp. = 130 °C for dyes **10** and **11** and Temp. = 110 °C for dye **4**), (pH = 2 for dyes** 10** and** 11** and pH = 8 for dye** 4**).

Dye	Time (min)	L*	a*	b*	C*	H^o^	E	Original pictures of the dyed fabrics
**4**	10	61.7	16.7	49.7	52.5	71.4	80.96	
	20	57.9	17.8	51.7	54.7	71	79.63	
	30	50.6	20.8	56	59.7	69.7	78.3	
	40	44.7	30.3	45.5	45.5	57.9	70.6	
	60	46.5	27.7	42.6	42.6	56.3	68.8	
**10**	10	46.6	12.1	32.7	34.9	69.7	58.2	
	20	46.3	12.1	31.9	34.1	69.3	57.5	
	30	44.5	12.8	41	42.9	72.6	61.8	
	40	53.97	11.5	40.4	41.9	74.3	68.4	
	60	56.3	11.3	30.3	32.4	69.1	55.1	
**11**	10	48.8	8.2	28.2	29.6	74.8	56.96	
	20	46.9	8.7	27.7	29.5	73.9	55.41	
	30	43.4	9	27.3	28.74	72.6	52.05	
	40	58.5	7	28.4	29.1	75.6	65.3	
	60	59.9	6.8	28.6	29.4	76.1	66.7

**Table 5:** Different shades have an impact on the measurements of L*, a*, C*, b*, H^o^, E, R%, and color strength for dyes **4,10** and **11** (Temp. = 130 °C for dyes **10** and **11** and Temp. = 110 °C for dye **4**), (pH = 2 for dyes **10** and **11** and pH = 8 for dye **4**), (time = 30 min for dyes **10** and** 11** and time = 40 min for dye **4**).

Dye	Shade	L*	a*	b*	C*	H^o^	E	Original pictures of the dyed fabrics
**4**	0.5%	64.5	18.3	29.9	34.5	70.6	72.9	
	1%	58.4	20.9	36.1	41.3	70.1	71.4	
	2%	52.7	22.6	37.7	43.9	63.6	68.7	
	3%	50.8	26.4	54.5	61.8	73.7	79.9	
	4%	41.8	27.5	56.7	63.3	71.9	75.9	
	5%	40.1	27.1	59.6	66.3	72.7	77.3	
	6%	39.2	28.8	63.1	69.6	73.5	79.7	
**10**	0.5%	77.2	6.4	32.7	33.5	79.4	83.9	
	1%	64.6	8.7	32.6	34.1	76.1	72.7	
	2%	55.3	8.9	32.9	34.3	71.6	65.5	
	3%	54.8	9.8	34.8	36.5	71.2	65.4	
	4%	48.9	13.1	35.3	37.65	69.5	61.7	
	5%	48.1	13.1	36.7	38.96	69.8	61.9	
	6%	46.8	14.6	40.8	43.3	70.1	63.8	
**11**	0.5%	70.8	6.1	24.3	25.1	78.8	75.1	
	1%	69.4	7.2	26.9	27.8	77.9	74.8	
	2%	67.1	7.8	27.9	29	76.1	73.1	
	3%	59.6	8.8	29.7	30.9	73.9	67.2	
	4%	58.5	9.3	31.6	33.0	73.6	67.1	
	5%	56.6	11.8	34.1	36.1	73.7	67.12	
	6%	54.7	12.6	36.2	38.3	79.9	77	

Based on Figs. 5, 6, 7, and 8 and Tables 2, 3, 4, 5 and 6, it was discovered that the factor that most affected the dyed fabric’s colour strength was time, in the case of dyes **10** and **11**, while the factor that most affected dye **4** was pH. Additionally, it was discovered from all the figures and tables that the synthetic dye number **4** works best for dyeing PET fabric.

**Table 6:** Summarize the optimized conditions.

Conditions	Dye **4**	Dye **10**	Dye **11**
pH	8	2	2
Temperature °C	110	130	130
Time (min)	40	30	30
Shade %	6	6	6

#### Color reproducibility

Numerous aspects of the dyeing process need to be regulated or measured to achieve reproducible dyeing, whether in a lab, pilot plant, or bulk setting. These include dye selection, moisture content, dye standardization, substrate weight, substrate preparation, substrate moisture content at weighing, water supply quality, dye and chemical weighing, alcohol content, dye bath pH, dye bath additives, the way of dispensing chemicals and colours, the profile of temperature and time, substrate dye ability, machine flow, and reversal sequence^75^. The laboratory dyeing process’s reproducibility was examined for several batches, and the findings are shown in Table 7. Table 6 summarizes the ideal dyeing process parameters for each dye separately and offers useful conditions for the laboratory dyeing process’s reproducibility. For all dyes, colour differences (∆E) between dyeing tests ranged from − 0.3 to 0.9. Technically, a colour difference of ± 1 is acceptable when using synthetic dyes^76^. The dyeing of polyester fabric with all dyes demonstrated good colour matching and reproducibility, as well as good tonal parameters, indicating that dyeing with these dyes is a viable technique, despite some colour degradation in solution depending on pH, time, and temperature.

**Table 7:** Differences in CIELab color coordinates and K/S values across all polyester dyeing.

Dyes	Batch	K/S values	L*	a*	b*	C*	∆E
**4**	1	26.7	50.3	20.2	55.4	58.9	–
	2	26.6	50.6	20.8	56	59.7	0.8
	3	26.8	49.7	21.3	56.5	60.4	0.7
**10**	1	8.4	54.8	11.9	37.5	39.3	–
	2	8.72	53.9	12.8	38.1	40.2	− 0.3
	3	8.9	54	11.5	40.4	41.9	0.9
**11**	1	7.84	58.5	9.3	31.6	33.0	–
	2	7.98	58.9	9.7	32.4	33.8	0.8
	3	7.74	59.6	8.8	29.7	30.9	0.1

#### Characterization of dyeing polyester

Table 8 shows the fastness parameters of the polyester dyeing material achieved with the prepared dyes at a dyeing temperature of 130 °C, shade 5%, and time of 30 min.

**Table 8:** Properties of dyeing polyester fabrics.

Dye number	Lightfastness	Washing fastness		Crock fastness
	After reduction clearing	Before reduction clearing	After reduction clearing	After reduction clearing
Dye **4**	6	3	4	4
Dye **10**	6–7	3–4	4–5	5
Dye **11**	6–7	4	5	5

#### Light, washing and crocking fastness

The lightfastness of any dyed polyester fibre is determined by a variety of parameters, the most important of which are the concentration of dye molecules in the fibre and the substituent groups found in the dye molecules. The substituent’s groups may interact with the fibre, raising or lowering the electron density surrounding it. The dyeing samples’ lightfastness properties range from very good to excellent (6 and 6–7/8) after reduction clearing for all dyes. The great lightfastness (6–7/8) of dyes **10** and **11** on polyester fabric could be attributed to a hydrazide-hydrazone moiety connected to the benzothiazole nucleus, which increases molecular weight. In general, the produced dyes were resistant to photo fading^77^.

The dyeing samples’ crock fastness properties range from very good (using dye **4**) to excellent (using dyes **10** and **11**) (5/5–5/5). This could also be ascribed to changes in the dyes’ structures, such as the presence of a hydrazide-hydrazone moiety nucleus in dyes (**10** and **11**), which results in a higher molecular weight for these dyes than dye **4**, as shown in Table 8.

**Fig. 9:**
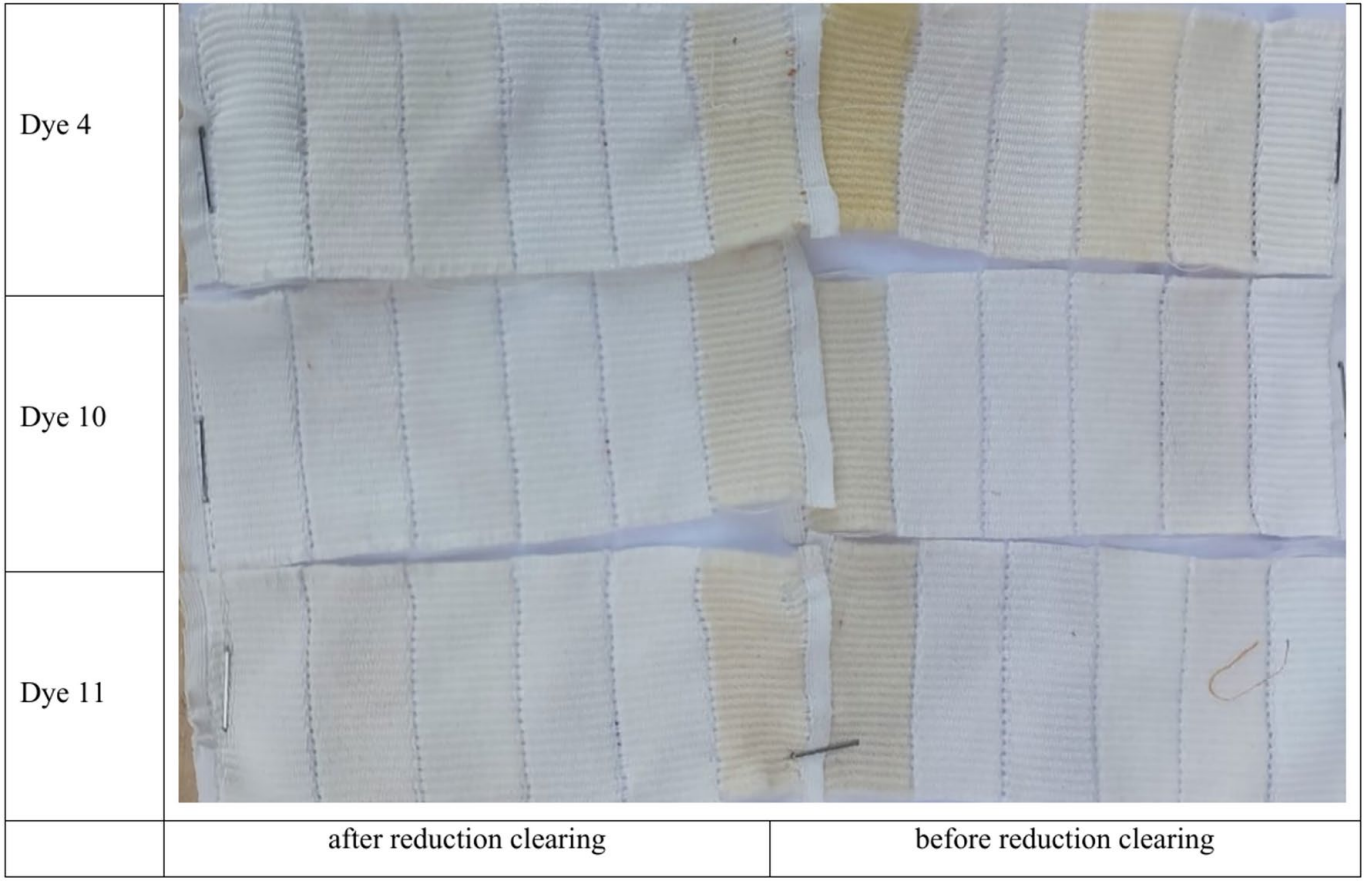
washing fastness for dyeing of polyester using dyes 4, 10, and 11 before and after reduction clearing.

The fastness-to-washing tests revealed that the dyes had acceptable ratings before reduction clearing Grades 3–4 and excellent ratings after reduction clearing Grades 4–5 for polyester fabric using dyes (**4** and **11**), with the exception of dye **10**, which had a rating of 5 and outperformed dyes **11** and **4**. The concordance of these results may be attributed to an increase in the large molecular size of the dye molecules (**10** and **11**), as well as the good diffusion of the dye molecules within the fabrics, as shown in Table 8 and Fig. 9^77^.

## Conclusions

To supplement our approach to the synthesis and characterization of novel disperse dyes, we have created three new disperse dyes and confirmed their chemical makeup. We then used these dyes to dye polyester fabrics at various pH values, times of 10–60 min, temperatures between 100 and 130 °C, and shades of 0.5–6. Because dye** 4** is smaller than dyes **10** and **11** in size and molecular weight, it is evident that dye 4 has a stronger colour than those two. The range of colours generated was sufficient in terms of colour levelness and shade depth, encompassing yellow, beige, light brown, reddish brown, and dark brown in different tones. These values generated the necessary dyeing levelness (light, washing, and crocking), fastness, and shade depth for dyes **4**,** 10**, and **11**. Importantly, the data underlines how to dye polyester materials with the best possible colour effects by using the right dyeing settings for the previously described dispersed dyes.

## Experimental

### Materials and instruments

All the solvents and chemicals were bought from Sigma-Aldrich in Missouri, USA, and didn’t require further purification. The 100% polyester fabric (140 gm/m^2^, plain weave, Yarn 48 * 150 denier, the threads are 110*80 warp in weft, width 66 inches, and thickness (0.39 mm/cm) was supplied by El-Nahawy Textile Company, Egypt. The starting substances, such as para-aminobenzophenone and m-dihydroxybenzene (resorcinol), were obtained from Sigma Aldrich. The other chemicals used for the experimental work include sodium nitrite (NaNO_2_), sodium acetate trihydrate (CH_3_COONa. 3H_2_O), sodium carbonate was acquired from Alpha Chemika, India, and concentrated hydrochloric acid (HCl) were collected from various resources such as SD Fine and CDH (Central Drug House). The organic solvents, such as ethanol and glacial acetic acid, were supplied by Lobachem without any further purification. We conducted thin layer chromatography (TLC) analysis using pre-coated silica gel aluminium plates (Macherey–Nagel) with DCM: MeOH (95:5%) as the eluting system. We monitored and visualised the reactions using UV light (254 nm). The melting point of synthetic dyes was determined with the SMP50 Digital Melting Point App provided by Bibby Scientific in Staffordshire, and by using solid potassium bromide pellets, we can determine the infrared spectra with a resolution of 4.0 cm⁻^1^, in a range of 4000–400 cm⁻^1^ on a Thermo Fisher Nicolet IS10 spectrophotometer. We used the JEOL-JAPAN instrument at 400 MHz and 101 MHz, respectively, to get ^1^H and ^13^C NMR spectra. Proton chemical shifts are labelled in parts per million (ppm), downfield from organosilicon compounds like tetramethyl silane (TMS, δ/ppm = 0.00) as an internal standard. We used the following abbreviations (or a combination thereof) to describe splitting patterns: s, singlet; d, doublet; t, triplet; q, quartet; m, multiplet; and br, broad. We used the residual protons (2.50, 3.33 ppm for ^1^H NMR and 39.9 ppm for ^13^C NMR) as internal references. We measured mass spectra with a Thermo Scientific GCMS model (Isq Lt) using the Thermo X-Calibur software (Shimadzu, Kyoto, Japan) at the Regional Centre for Mycology and Biotechnology (RCMB), Al-Azhar University, Nasr City, Cairo, Egypt. Elemental studies were conducted at the Regional Centre for Microbiology and Biotechnology, Al-Azhar University, Cairo, Egypt. The results have an accuracy of within 0.4%. All the recently developed compounds melting points (MPs) were measured using open capillaries and a digital Gallen Kamp MFB-595 analyzer. The optical characteristics of the specimens were measured and studied using a UV–visible spectrometer (UV–Vis, Jenway Model 6700). The process of dyeing was finished using laboratory AHIBA infrared dyeing equipment. A datacolor 850 was used to determine the colorimetric properties of the dyed fabric. Finally, lightfastness was tested using a SUNTEST CPS + (Heraeus, Milan, Italy) with a xenon lamp from Atlas.

### Preparation of new disperse dyes

#### Synthetic procedure for (*E*)-(4-((2,4-dihydroxyphenyl)diazenyl)phenyl)(phenyl)meth- anone 4

To generate a diazonium salt solution** 2**, a portion-wise addition of sodium nitrite (0.01 mol) was added to a cool solution of 4-aminobenzophenone **1** (0.01 mol) in a mixture of 35% HCl (10.5 mL) and water (40 mL) for 30 min. Subsequently, compound **3** in ethanol and sodium acetate trihydrate (0.02 mol) gradually received the reaction mixture. After one hour of continuous stirring at 5 °C and an additional hour at the ambient temperature, the reaction generated a crimson precipitate that was filtered and carefully cleaned with cold water. Following a period of air drying, compound **4** was gathered.

Yield 90%; red powder; m.p.:198–200 °C, FT-IR (KBr, ν_max_/cm^−1^); 3278 (OH), 3059 (Ar–CH), 1623 (C = O), and 1592 (N = N)^1^H NMR (400 MHz, DMSO-*d*6) δH/ppm:12.36,10.74 (2 s, 2H, 2OH, exchangeable by D_2_O), 8.00–7.95 (m, 2H, Aromatic-H), 7.89–7.85 (m, 2H, Aromatic-H), 7.78–7.73 (m, 2H, Ar–H), 7.71–7.65 (m, 3H, Ar–H), 7.57 (dd, J = 8.2, 6.9 Hz, 1H, resorcinol-H), 6.51 (dd, J = 8.9, 2.5 Hz, 1H, resorcinol-H), 6.37 (d, J = 2.5 Hz, 1H, resorcinol-H)^13^C NMR (100 MHz, DMSO-*d*_6_) δ:195.51(C = O), 164.56, 158.09, 153.48, 137.74, 137.46, 133.39, 133.24, 131.51, 130.05, 129.88,129.09,122.04,110.21,103.49.EI-MS (m/z):318 [%]: [M + , (10.16%] Anal. Calcd for C_19_H_14_N_2_O_3_ (318.33): C, 71.69; H, 4.43; N, 8.80; Found: C, 71.95; H, 4.31; N, 8.70%.

#### Synthesis of 2-benzothiazolyl acetohydrazide 9

2-aminothiophenol **5** (0.01 mol) and cyanoacetohydrazide **8** (0.01 mol) were added together and heated to 60 °C for an hour, followed by a 15-min reflux in a solution of methanol and glacial acetic acid (5 ml each). The solution was cooled, and then ether was added. The precipitate that formed was then filtered out and crystallized; m.p.156–157°C (lit.^64^ 155–157 °C).

#### General procedure for the synthesis of azo dyes 10 and 11

Add a solution of drops of glacial acetic acid and 0.01 mol of benzophenone **4** in 15 mL of ethanol to a mixture of 0.01 mol of hydrazide in 15 mL of ethanol, and reflux for three hours. Allow the reaction mixture to cool to room temperature once thin-layer chromatography (TLC) analysis indicates that the reaction is complete. Next, use vacuum-assisted evaporation to reduce the ethanol volume. Crude products can be obtained by filtering the precipitate and washing it with alcohol. Ultimately, the target compound has been generated through the recrystallization of the crude compounds with appropriate solvents.

#### *N*'-((*E*)-(4-((***E***)-(2,4-dihydroxyphenyl)diazenyl)phenyl)(phenyl)methylene)benzo[*d*]thiazole-2-carbohydrazide 10

Yield 76%; caffee powder; m.p. > 300 °C, FT-IR (KBr ,ν_max_/cm^−1^); 3381 (2OH), 3198 (NH), 3060 (Ar–CH), 1649(C=O), 1632 (C=N), and 1596 (N=N)^1^H NMR (400 MHz, DMSO-*d*_6_) δ: 12.20 (s, 2H, 2OH, exchangeable by D_2_O), 10.41 (s, 1H, NH, exchangeable by D_2_O), 8.18 (dd, J = 7.9, 1.3 Hz, 2H), 8.08 (d, J = 8.0 Hz, 1H, Ar–H), 7.96 (d, J = 8.6 Hz, 2H, Ar–H), 7.86 (d, J = 8.6 Hz, 2H, Ar–H), 7.75 (d, J = 7.0 Hz, 1H, Ar–H), 7.72–7.64 (m, 2H, Ar–H), 7.63–7.50 (m, 4H, Ar–H), 6.51 (dd, J = 8.9, 2.5 Hz, 1H, resorcinol-H), 6.37 (d, J = 2.4 Hz, 1H, resorcinol-H)^13^C NMR (100 MHz, DMSO-*d*_6_) δC/ppm: 164.04 (C=O), 159.29 (C=N),158.60, 158.09, 153.43, 153.24, 152.15, 150.32, 137.42, 136.11, 133.37, 133.25, 132.70, 131.51, 130.63, 130.04, 129.09, 127.50, 127.16, 124.35, 123.32, 122.00, 110.28, 103.50. EI-MS (m/z): 493 (M + ;10.23%).Anal. Calcd for C_27_H_19_N_5_O_3S_ (493.54): C, 65.71; H, 3.79; N, 14.19;Found: C, 65.60; H, 3.06; N, 14.08%.

#### 2-(benzo[*d*]thiazol-2-yl)-*N*'-((*E*)-(4-((*E*)-(2,4-dihydroxyphenyl)diazenyl)phenyl)(phenyl) methylene)acetohydrazide 11

Yield 78%; brown powder;m.p.: > 300 °C, FT-IR (KBr, ν_max_/cm^−1^); 3188 (OH), 3059 (Ar–CH), 1647 (C=O), 1618 (C=N), and 1595 (N=N). ^1^H NMR (400 MHz, DMSO-*d*_6_) δH/ppm: 10.73,12.37 (2s, 2H, 2OH, exchangeable by D_2_O), 10.55 (s, 1H,NH, exchangeable by D_2_O), 8.05 (d, J = 7.9 Hz, 1H, Ar–H), 7.95 (dd, J = 14.4, 8.1 Hz, 3H, Ar–H), 7.87 (dd, J = 8.3 Hz, 2H, Ar–H), 7.75 (dd, J = 6.8 Hz, 2H, Ar–H), 7.68 (t, J = 8.1 Hz, 2H, Ar–H), 7.56 (t, J = 7.5 Hz, 2H, Ar–H), 7.51–7.44 (m, 1H, Ar–H), 7.40 (t, J = 7.5 Hz, 1H,resorcinol-H), 6.51 (dd, J = 8.8, 2.5 Hz, 1H, resorcinol-H), 6.37 (d, J = 2.4 Hz, 1H, resorcinol-H), 4.13 (s, 2H, CH_2_)^13^C NMR (100 MHz, DMSO-*d*_6_) δC/ppm: 175.49 (C=O), 168.70 (C=N), 166.35, 166.35, 164.94, 164.56, 158.09, 153.49, 137.73, 135.77, 133.24, 131.50, 130.04, 129.08, 126.49, 125.46, 122.73, 122.46, 122.05, 110.21, 103.51, 39.10(CH_2_). EI-MS (m/z): 507 (M + ;6.72%), Anal. Calcd for C_28_H_21_N_5_O_3_S (507.57):C, 66.26; H, 4.17; N, 13.80;Found: C, 66.15; H, 4.06; N, 13.71%.

#### Preparation of polyester fabric

The polyester was cleaned and then left for 30 min at 80 °C in an aqueous solution containing 2 g of nonionic detergent and 2 g of sodium carbonate. Through this treatment, the raw polyester was free of any possible manufacturing contaminants and oils^78^.

#### Dye dispersion preparation and dyeing

**Fig. 10:**
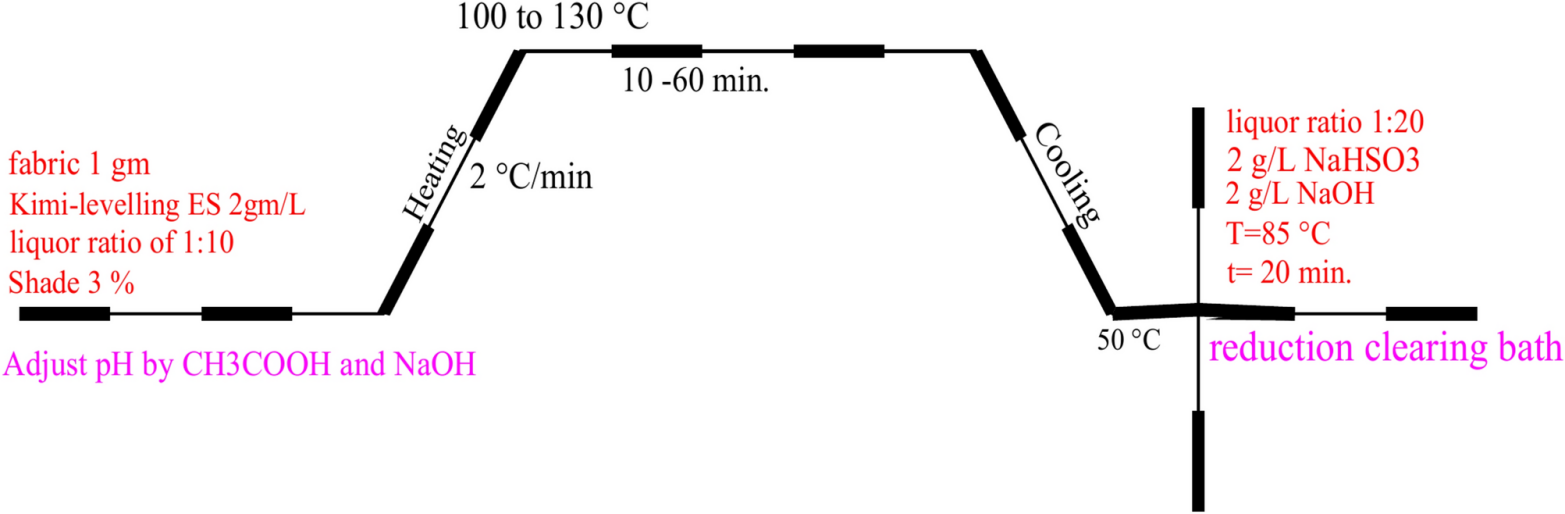
Dyeing curve for polyester using dyes **4, 10**, and **11**.

AHIBA’s laboratory environment Polyester was colourized using infrared dyeing equipment. A liquor ratio of 1:10, with 3% shade, and Kimi-levelling ES 2 g/L (a levelling and dispersion agent based on oleic acid polythene glycol 400 ester) supplied by an Egyptian chemical firm was used (the levelling and dispersing agent affects both the rate and amount of dispersed dye uptake on hydrophobic fibres by increasing the dyes’ aqueous solubility and affinity for the aqueous phase)^71,72^. The previously mentioned dispersion dye was prepared. Acetic acid and sodium hydroxide were used to lower and raise the pH of the dye baths to 2, 4, 6, and 8^79^. The temperature of the dye bath is raised gradually at a rate of 2 °C/min while the polyester textiles are submerged in it. Higher temperatures of 100–130 °C are reached. Depending on the required temperature, the dye bath is maintained at this high temperature for 10, 20, or 3 min^80,81^. The reduction-clearing procedure was performed in an aqueous solution containing 2 g/L sodium hydrogen sulfite and 2 g/L sodium hydroxide with a liquor ratio of 1:20 for 20 min at 85 °C, as can be observed in Fig. 10. After being thoroughly cleaned with tap water, they were neutralized in a separate bath with 1 g of acetic acid per liter for five minutes at 60 °C, 82 °C. After being thoroughly cleaned with tap water, they were neutralized in a separate bath with 1 g of acetic acid per litre for 5 min at 60 °C^77,83^.

#### Color yield assessment

The colorimetric properties of coloured fabric samples were determined using a Datacolor 850 spectrophotometer, along with the CIELAB colour strength values (K/S) and colour coordinates (L*, a*, b*, C*, and Ho)^24^. The K/S value was used to determine the surface colour strength of dyed fabric samples, whereas the reflectance value at a maximum wavelength was used to estimate the colour strength of the fabric^84^. This study used spectral data from the dyed materials to assess the impact of the nature of the different substituents on dyeing behaviour, colour hue, and depth. Utilising the Kubelka–Munk function f(R), which characterises the absorption "K" and scattering "S" of light, the optical characteristics of the samples were assessed. The dye materials play a major role in determining the absorption of "K." Simultaneously, the substrate affects the scattering "S. Equation (1), which is based on the wavelength of light, can be used to compute the reflectance (R) of a thick, opaque material with constant values of "K" and "S" using the Kubelka–Munk theory^25^.

1 $${\text{K}}/{\text{S }} = \, ({1} - {\text{R}})^{{2}} /{\text{2R}}$$

Equation (2) was used to calculate the colour difference (ΔE) of the polyester fabric samples that were dyed^68^.

2 $$\Delta {\text{E}} = \left[ {\left( {\Delta {\text{ L}}} \right)^{{2}} + \left( {\Delta {\text{b}}} \right)^{{2}} + \left( {\Delta {\text{a}}} \right)^{{2}} } \right]^{{{1}/{2}}}$$

3 $${\text{C}}* = \left( {{\text{a}}^{{2}} + {\text{b}}^{{2}} } \right)^{{{1}/{2}}}$$

4 $${\text{H}}^{{\text{o}}} = {\text{ tan}}^{ - 1} {\text{a}}/{\text{b}}$$

Lightness (L*), chroma (C*), and hue angle (H^o^) from 0 to 360°; a*, whose value indicates the degree of greenness (negative) and redness (positive); and b*, whose value indicates the degree of blueness (negative) and yellowness (positive). The polyester fabric’s colour hues of all dispersed dyes moved in a yellowish direction, as shown by the positive values of the yellow-blue axis, or b*^85,86^.

#### Evaluation of dyeing of polyester fabric

Wash fastness: ISO105-C10:C:2010 (60 °C) for 30 min on multifiber neighbouring cloth following and before reduction clear bath, rating 1–5. Light fastness: AATCC TM16, Rating 1–8. Crock fastness: Crockmetre using the standard ISO105-X12-2016 method, rating 1–5.

## Supplementary Information

Supplementary Information.

## Data Availability

The data used and analyzed during the current study are available from the corresponding authors upon reasonable request.
