# Supplementary material for: Novel Azo dyes containing a hydrazide-hydrazone moiety for dyeing polyester fabric
Source: Sci Rep. 2025 Feb 5;15:4360. doi: 10.1038/s41598-024-83565-3 (PMC11799431; doi:10.1038/s41598-024-83565-3)
Supplement: Supplementary file 1 — Supplementary Information. [file 41598_2024_83565_MOESM1_ESM.pdf]

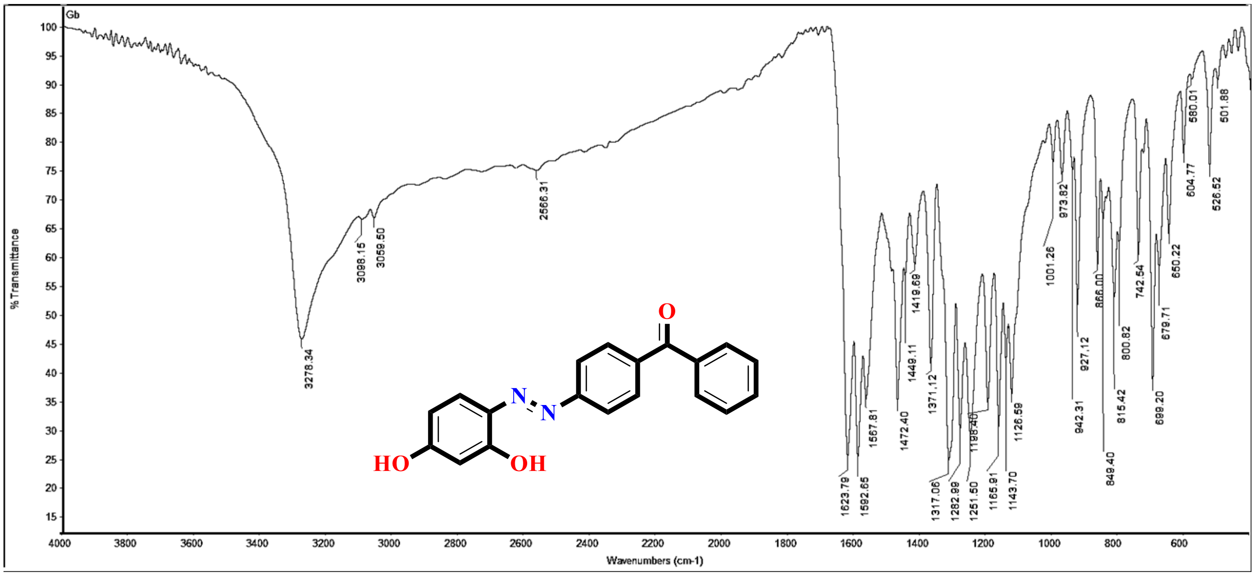

Fig. S1: FT-IR of Compound 4

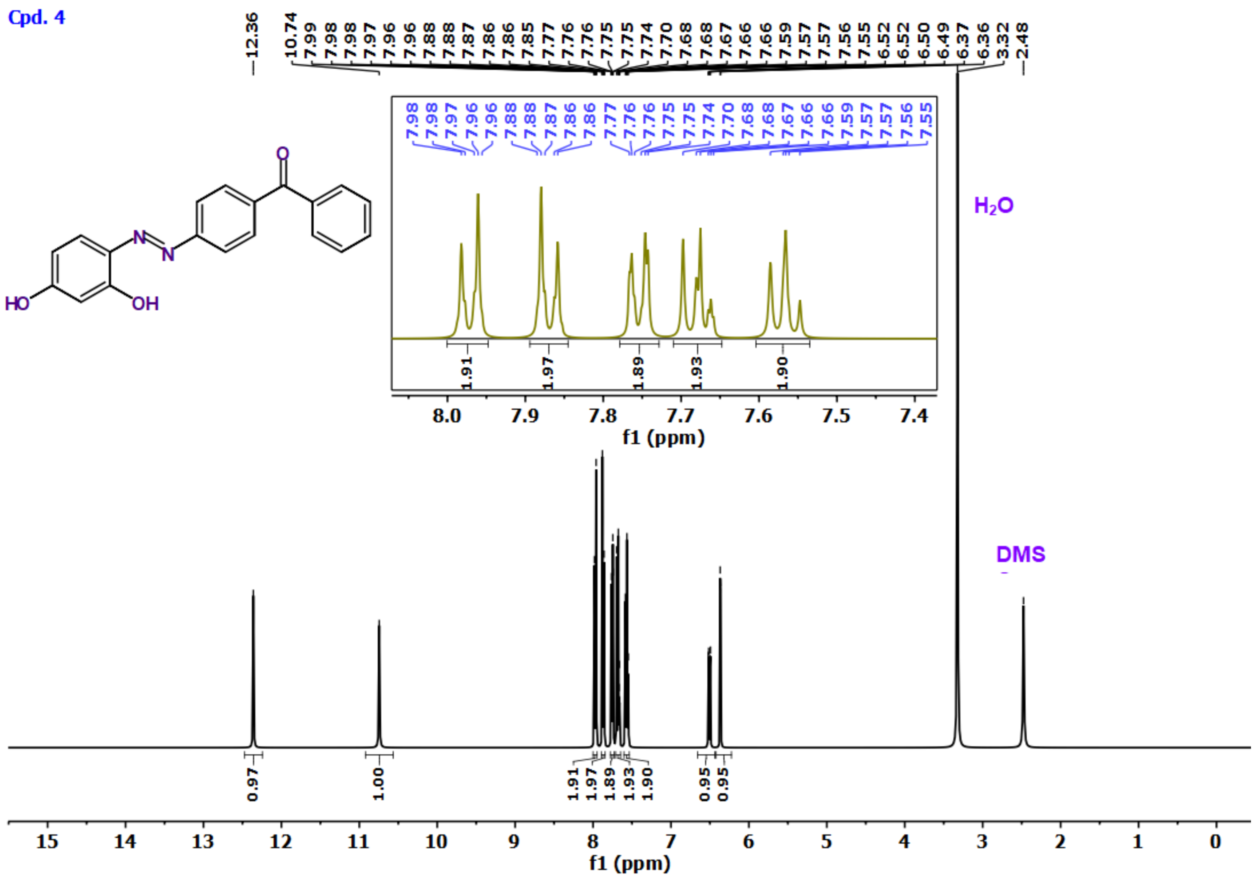

Fig. S2: ^{1}H NMR (DMSO-d$_6$) of Compound 4

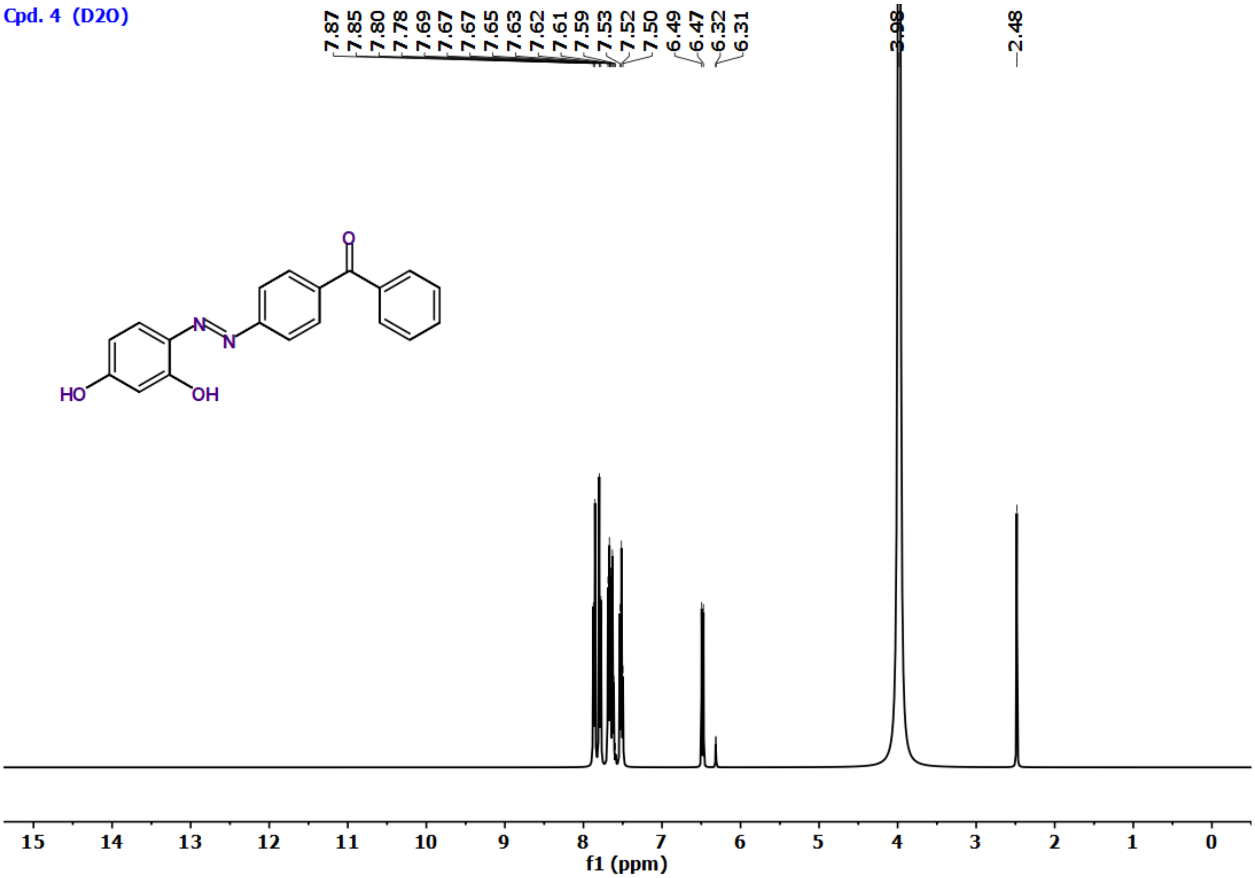

Fig. S3: ^{1}H NMR (DMSO-D_2O) of Compound 4

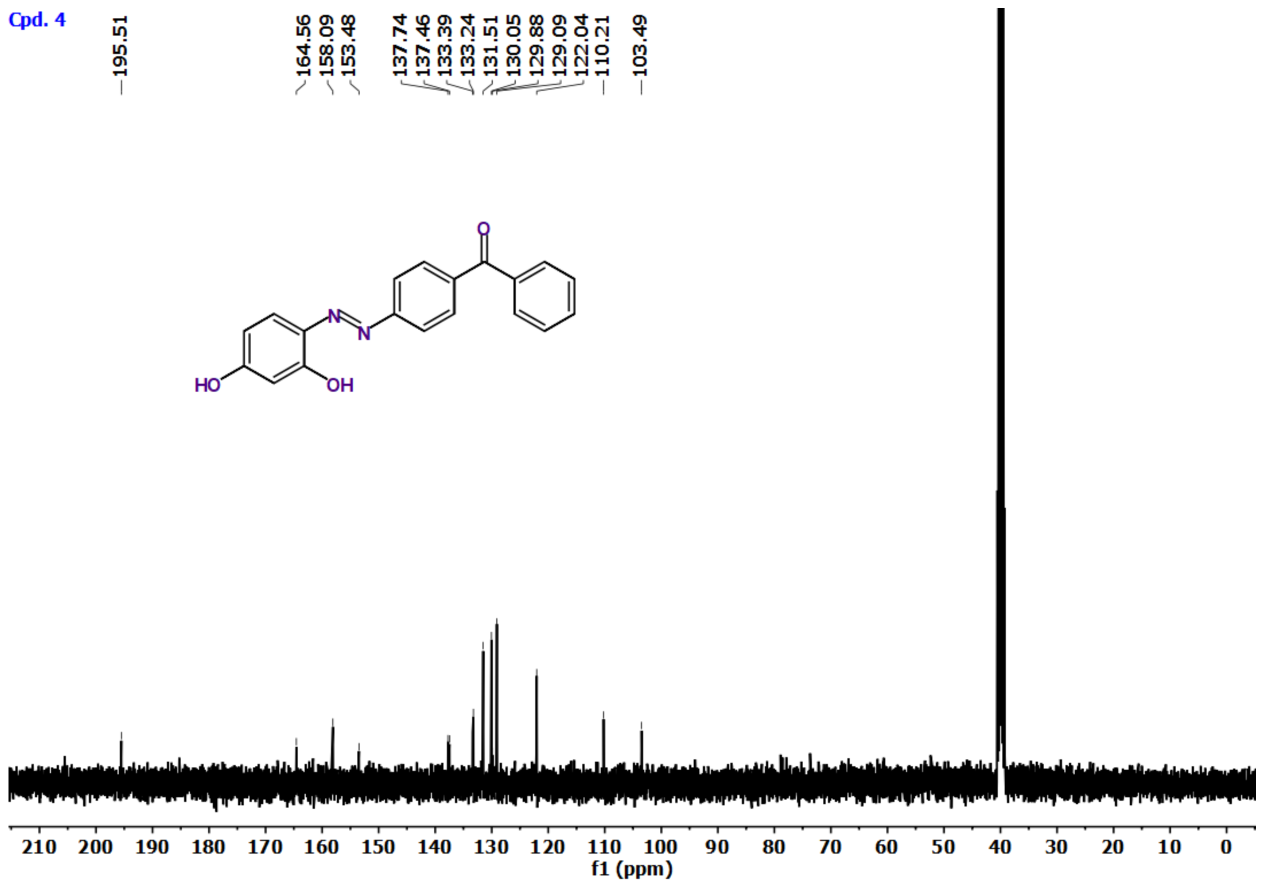

Fig. S4: ^{13}C NMR (DMSO-d_6) of Compound 4

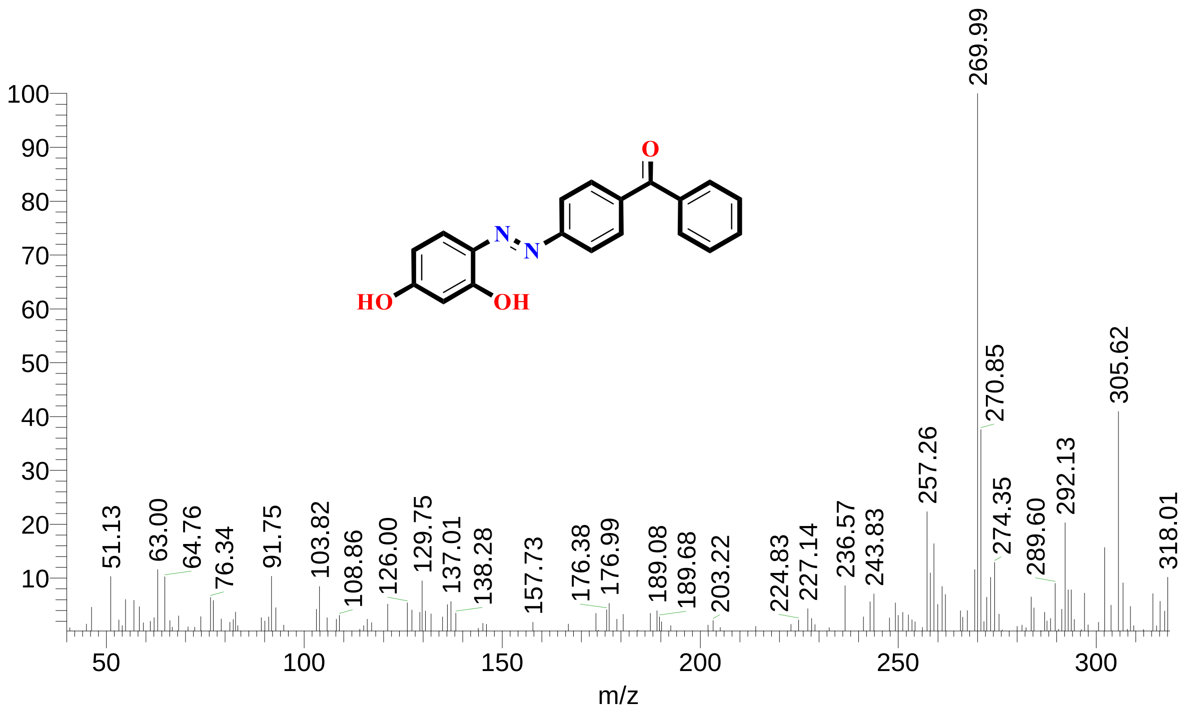

Fig. S5: Mass Spectrum of Compound 4

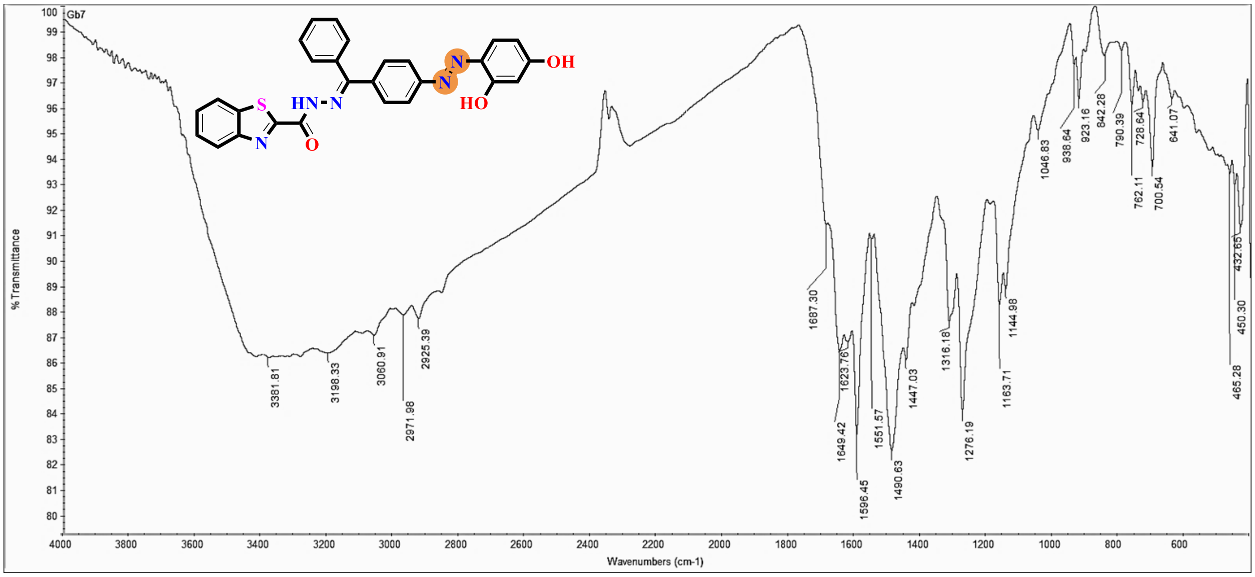

Fig. S6: FT-IR of Compound 10

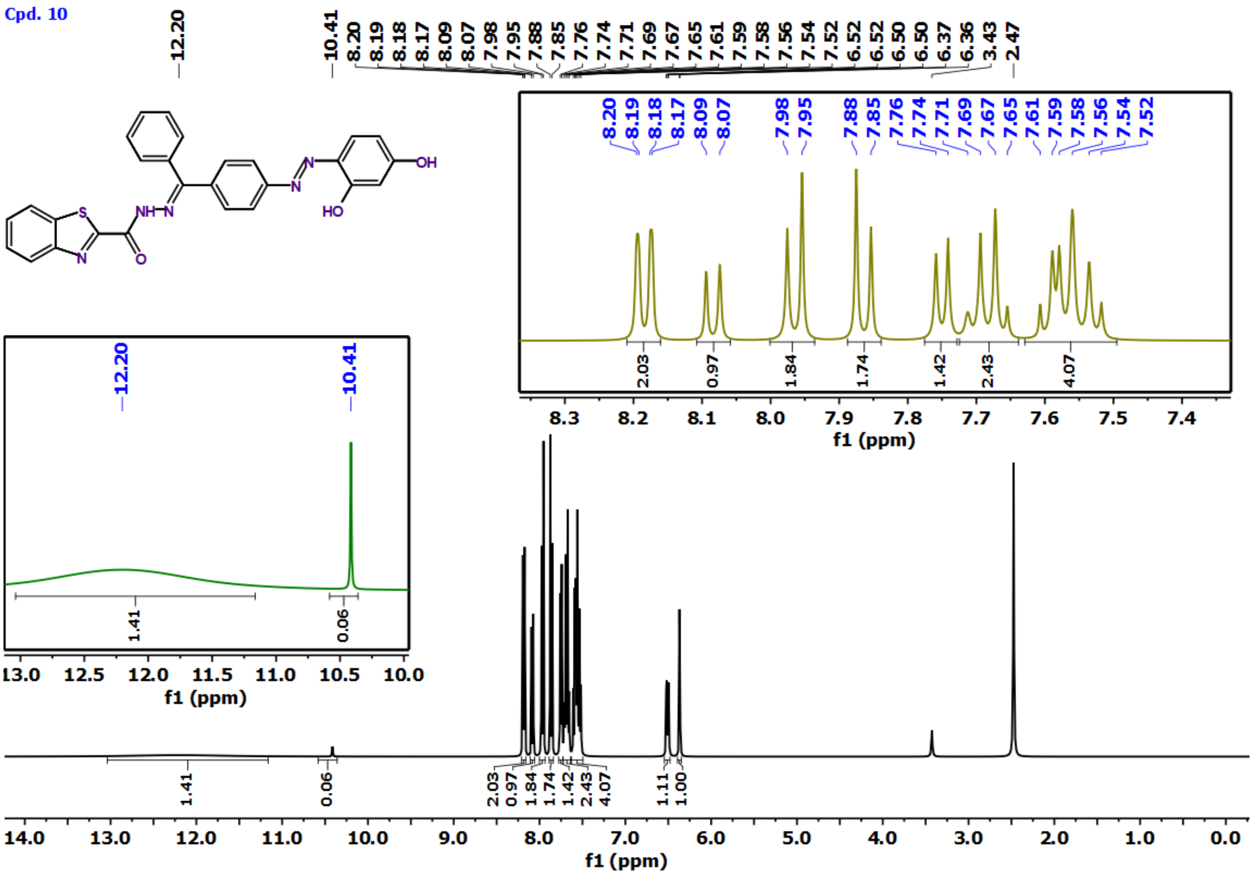

Fig. S7: ^{1}H NMR (DMSO-d_6) of Compound 10

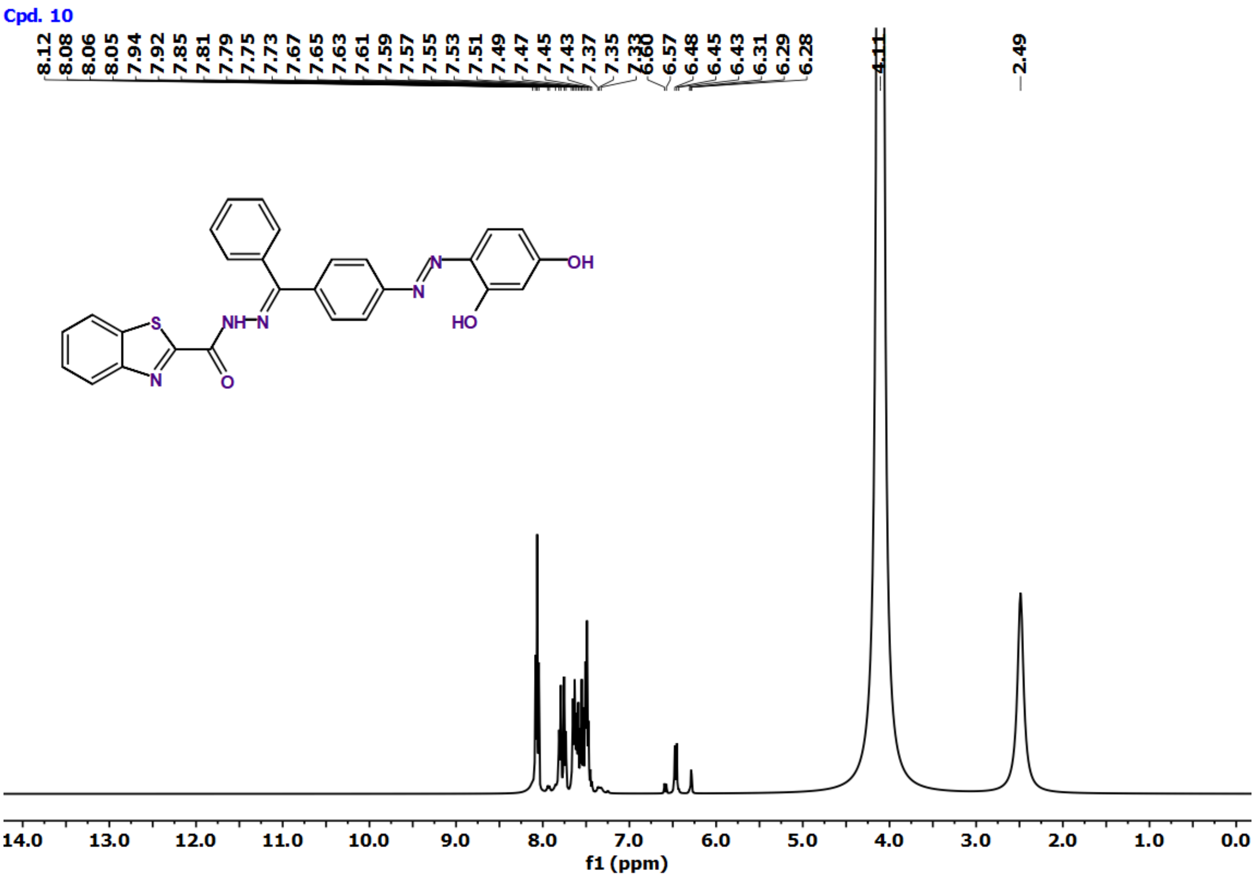

Fig. S8: ^{1}H NMR (DMSO-D_2O) of Compound 10

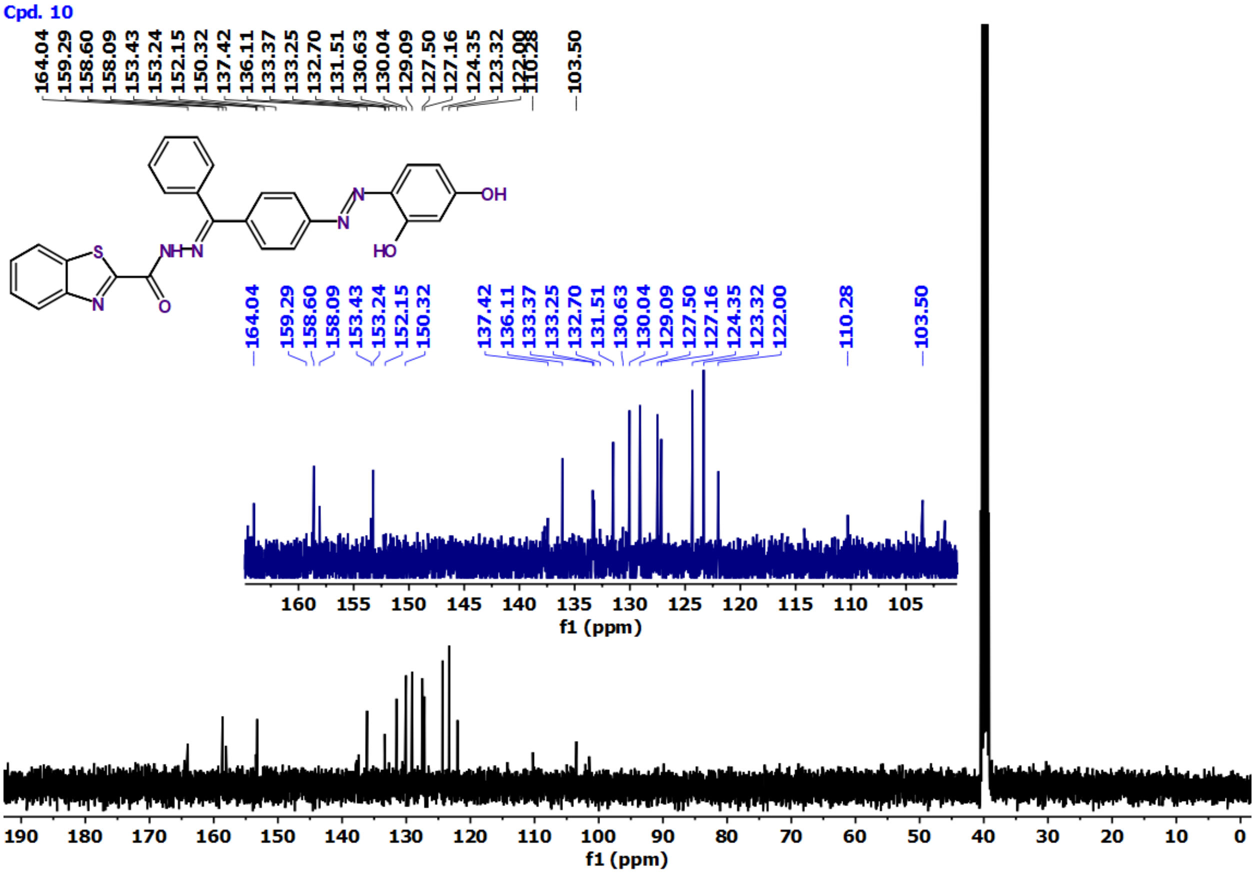

Fig. S9: ^{13}C NMR (DMSO-d$_6$) of Compound 10

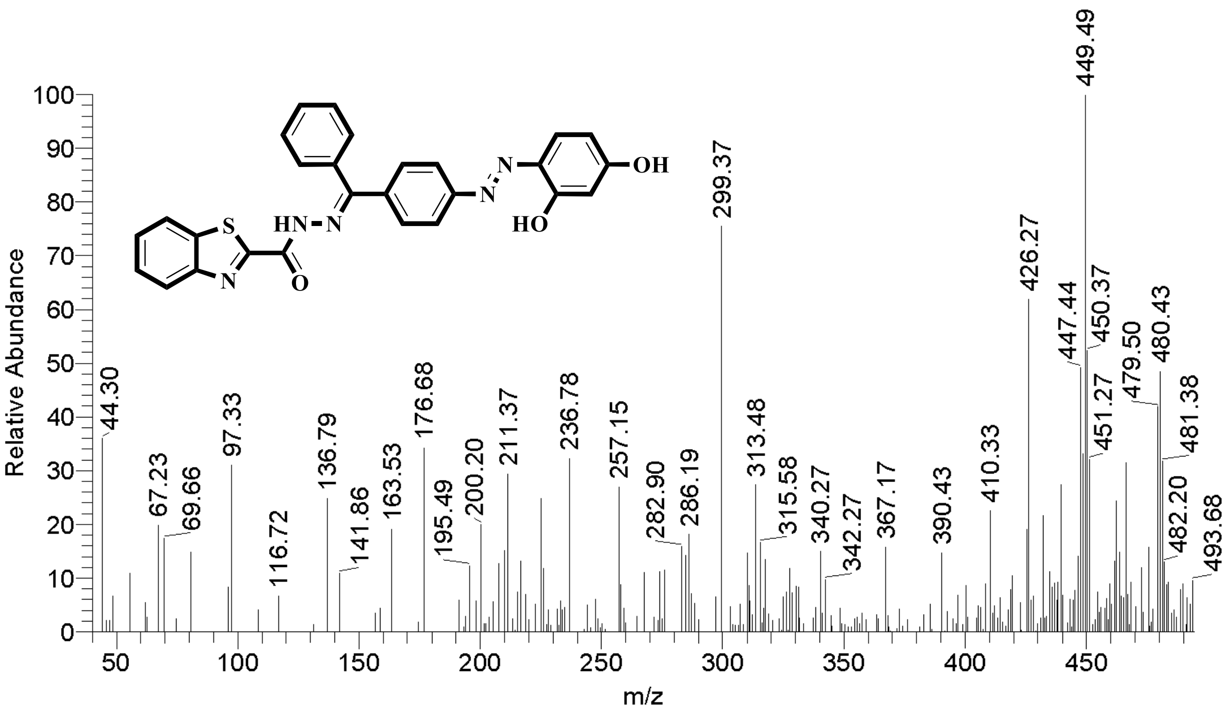

Fig. S10: Mass Spectrum of Compound 10

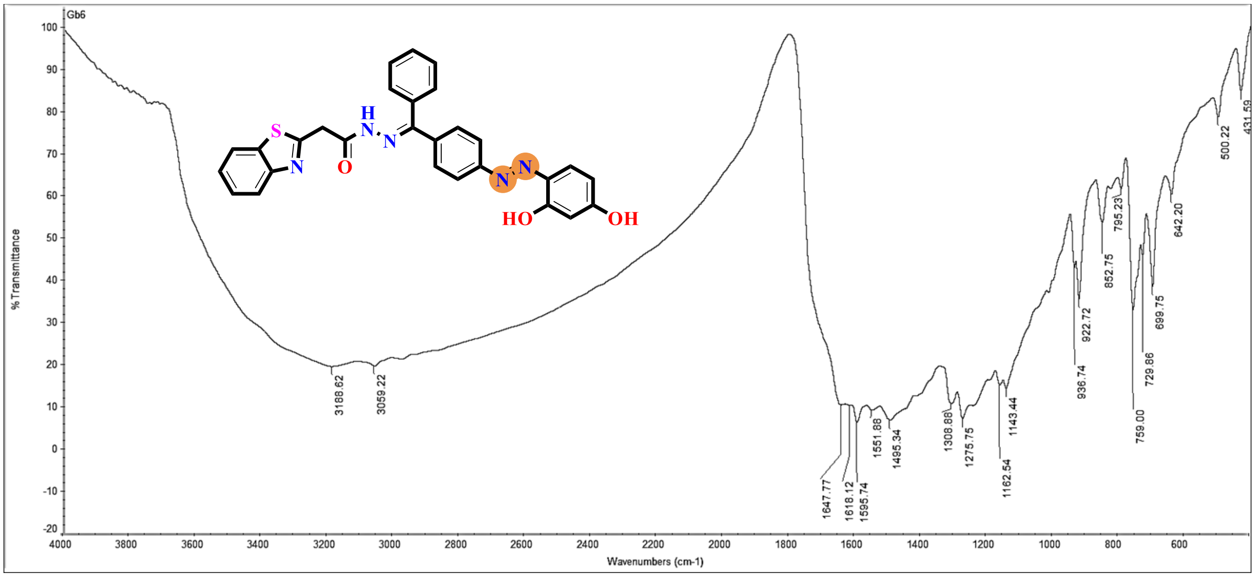

Fig. S11: FT-IR of Compound 11

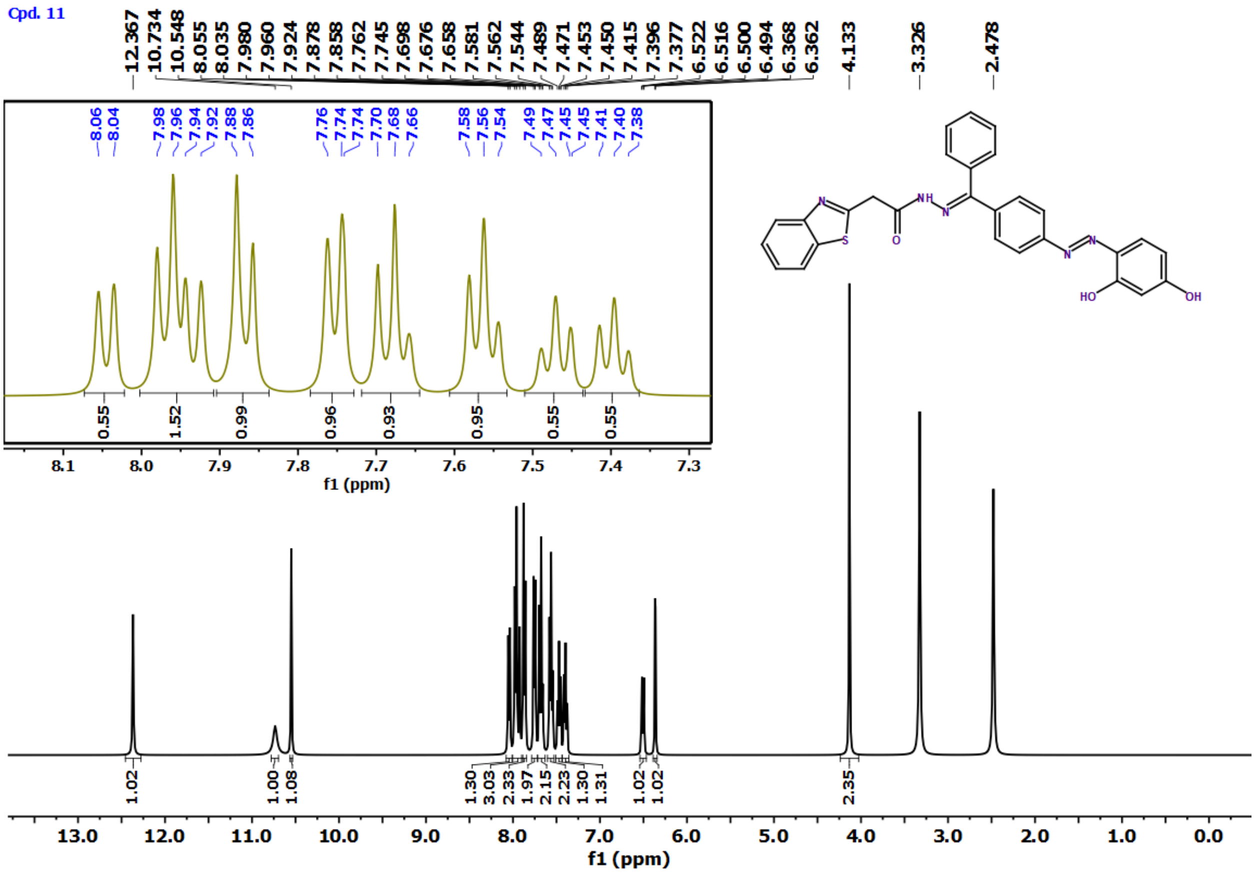

Fig. S12: ^{1}H NMR (DMSO-d_6) of Compound 11

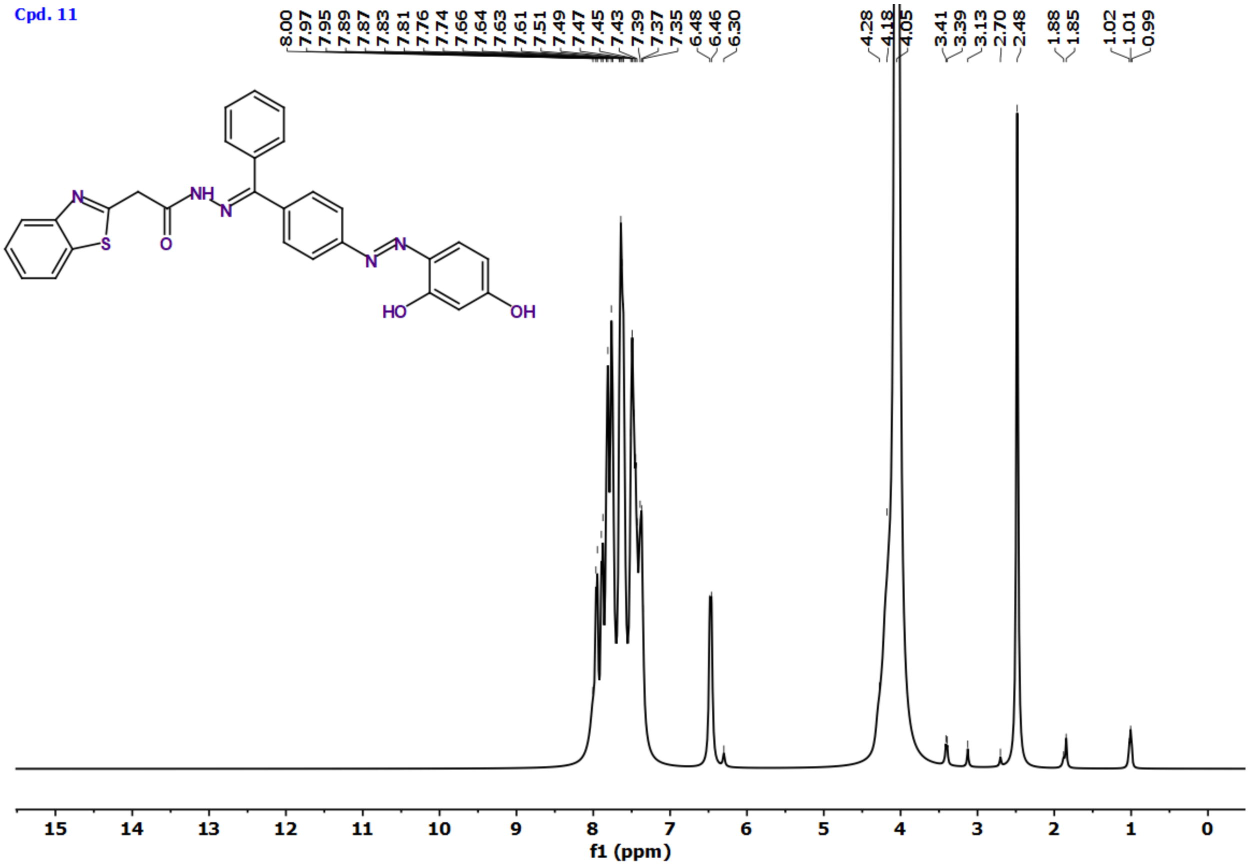

Fig. S13: ^{1}H NMR (DMSO-D_2O) of Compound 11

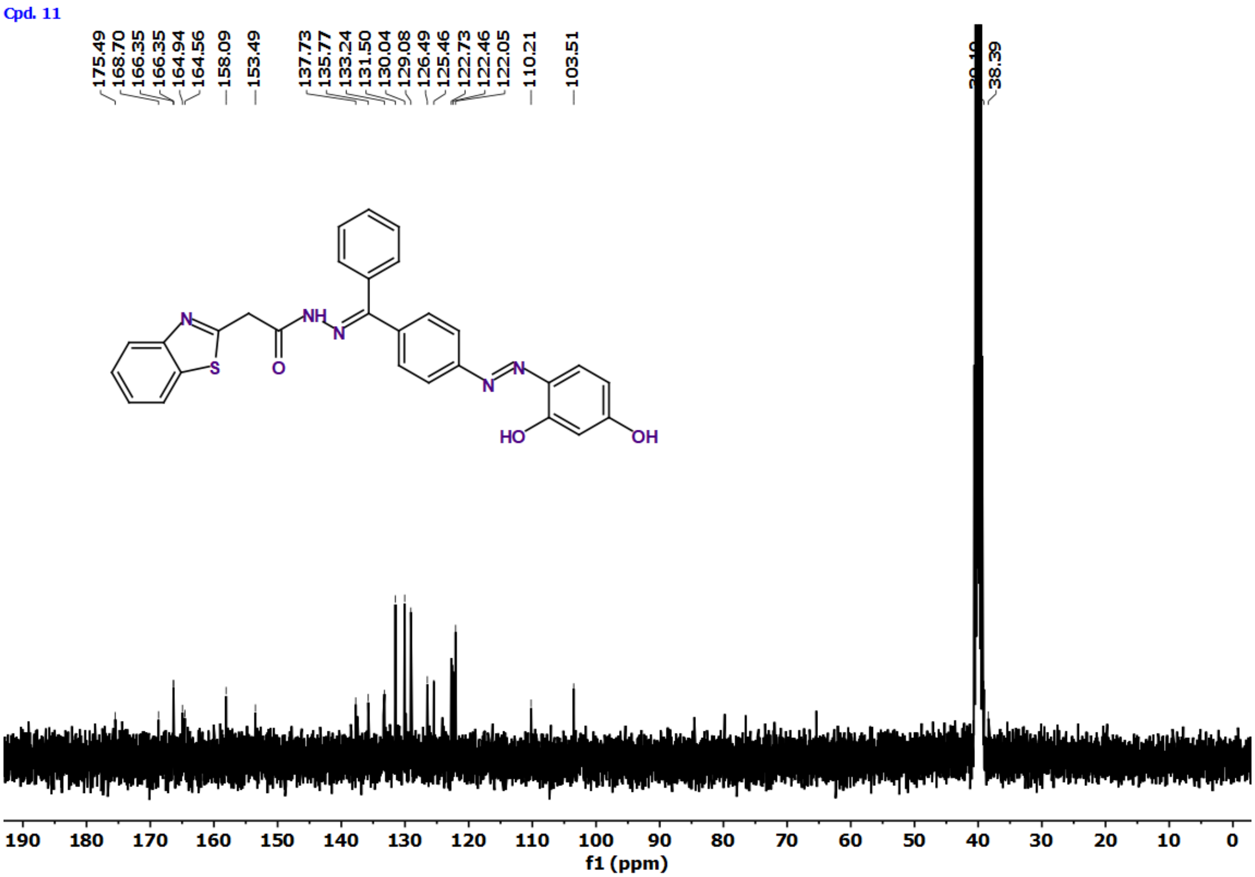

Fig. S14: ^{13}C NMR (DMSO-d_6) of Compound 11

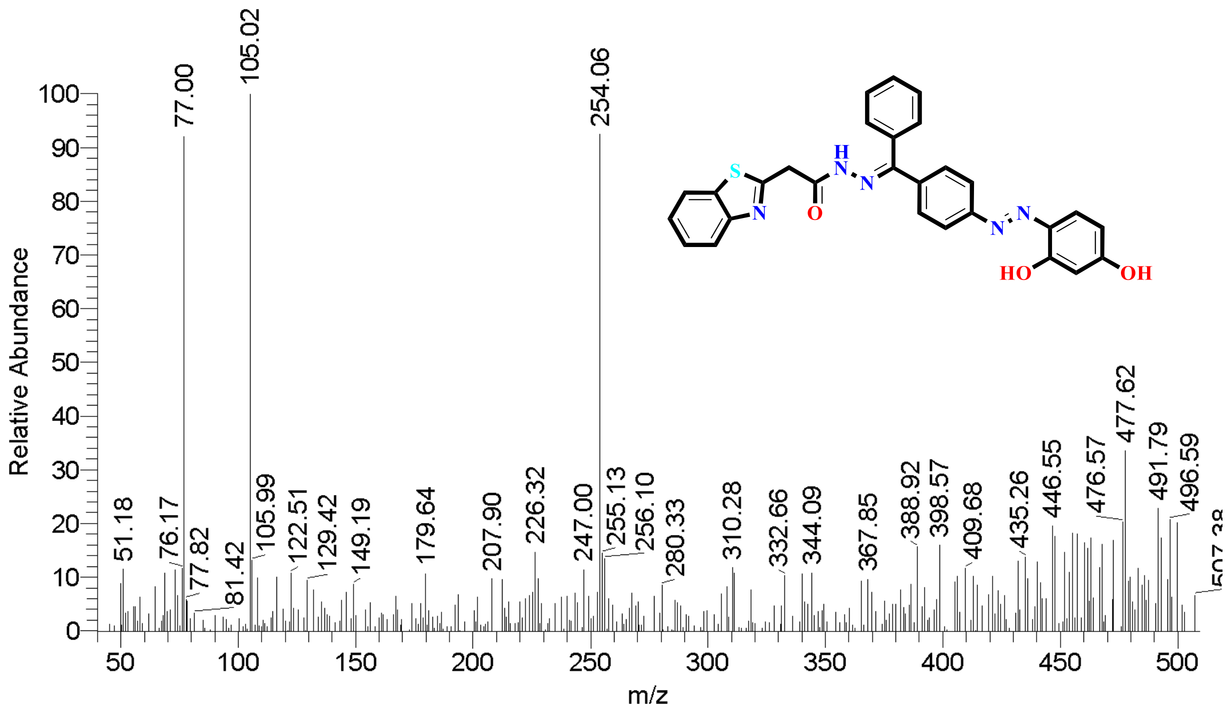

Fig. S15: Mass Spectrum of Compound 11
